# Behavioral pattern separation and cognitive flexibility are enhanced in a mouse model of increased lateral entorhinal cortex-dentate gyrus circuit activity

**DOI:** 10.3389/fnbeh.2023.1151877

**Published:** 2023-06-01

**Authors:** Sanghee Yun, Ivan Soler, Fionya H. Tran, Harley A. Haas, Raymon Shi, Grace L. Bancroft, Maiko Suarez, Christopher R. de Santis, Ryan P. Reynolds, Amelia J. Eisch

**Affiliations:** ^1^Department of Anesthesiology and Critical Care Medicine, Children's Hospital of Philadelphia, Philadelphia, PA, United States; ^2^Perelman School of Medicine, University of Pennsylvania, Philadelphia, PA, United States; ^3^University of Pennsylvania, Philadelphia, PA, United States; ^4^Department of Neuroscience and Mahoney Institute for Neurosciences, Perelman School of Medicine, University of Pennsylvania, Philadelphia, PA, United States

**Keywords:** fan cell, location discrimination, touchscreen based operant learning, TRIP8b, HCN

## Abstract

Behavioral pattern separation and cognitive flexibility are essential cognitive abilities that are disrupted in many brain disorders. A better understanding of the neural circuitry involved in these abilities will open paths to treatment. In humans and mice, discrimination and adaptation rely on the integrity of the hippocampal dentate gyrus (DG) which receives glutamatergic input from the entorhinal cortex (EC), including the lateral EC (LEC). An inducible increase of EC-DG circuit activity improves simple hippocampal-dependent associative learning and increases DG neurogenesis. Here, we asked if the activity of LEC fan cells that directly project to the DG (LEC → DG neurons) regulates the relatively more complex hippocampal-dependent abilities of behavioral pattern separation or cognitive flexibility. C57BL/6J male mice received bilateral LEC infusions of a virus expressing shRNA TRIP8b, an auxiliary protein of an HCN channel or a control virus (SCR shRNA). Prior work shows that 4 weeks post-surgery, TRIP8b mice have more DG neurogenesis and greater activity of LEC → DG neurons compared to SCR shRNA mice. Here, 4 weeks post-surgery, the mice underwent testing for behavioral pattern separation and reversal learning (touchscreen-based location discrimination reversal [LDR]) and innate fear of open spaces (elevated plus maze [EPM]) followed by quantification of new DG neurons (doublecortin-immunoreactive cells [DCX+] cells). There was no effect of treatment (SCR shRNA vs. TRIP8b) on performance during general touchscreen training, LDR training, or the 1st days of LDR testing. However, in the last days of LDR testing, the TRIP8b shRNA mice had improved pattern separation (reached the first reversal more quickly and had more accurate discrimination) compared to the SCR shRNA mice, specifically when the load on pattern separation was high (lit squares close together or “small separation”). The TRIP8b shRNA mice were also more cognitively flexible (achieved more reversals) compared to the SCR shRNA mice in the last days of LDR testing. Supporting a specific influence on cognitive behavior, the SCR shRNA and TRIP8b shRNA mice did not differ in total distance traveled or in time spent in the closed arms of the EPM. Supporting an inducible increase in LEC-DG activity, DG neurogenesis was increased. These data indicate that the TRIP8b shRNA mice had better pattern separation and reversal learning and more neurogenesis compared to the SCR shRNA mice. This study advances fundamental and translational neuroscience knowledge relevant to two cognitive functions critical for adaptation and survival—behavioral pattern separation and cognitive flexibility—and suggests that the activity of LEC → DG neurons merits exploration as a therapeutic target to normalize dysfunctional DG behavioral output.

## Introduction

Successful adaptation and survival demand the ability to discriminate stimuli (learn which stimulus is associated with a reward) and to be flexible (change behavior when reward conditions change) (Yassa and Stark, 2011; Dajani and Uddin, 2015; Prado et al., 2017; Cayco-Gajic and Silver, 2019). An example of stimuli discrimination is behavioral pattern separation, where a subject learns over time which stimulus (S) is associated with a meaningful outcome, such as reward delivery (S+). When stimuli or episodes are very similar, and thus the cognitive load is high (Yassa et al., 2011; Bekinschtein et al., 2013; Kent et al., 2015a,b; Kassab and Alexandre, 2018), this discrimination learning can be considered a behavioral readout of *pattern separation*, a process by which the brain avoids confusion between similar memories (Rolls and Kesner, 2006; Schmidt et al., 2012; Kesner and Rolls, 2015; Anacker and Hen, 2017; Severa et al., 2017; Cayco-Gajic and Silver, 2019). A key component of flexible learning is reversal learning, where a subject adapts its behavior when outcome conditions change (as when S+ becomes S-). Reversal learning is a behavioral readout of *cognitive flexibility* which, together with strategy shifting, allows successful adaptation to a changing world (Bissonette and Powell, 2012; Izquierdo et al., 2017). Many studies suggest that behavioral pattern separation and cognitive flexibility engage and rely on the integrity of hippocampal circuitry (Leutgeb et al., 2007; Clelland et al., 2009; Yassa and Stark, 2011; Burghardt et al., 2012; Swan et al., 2014; Anacker and Hen, 2017; Amer and Davachi, 2023). Far less is known about the role of a brain region that provides direct input to the DG: the entorhinal cortex (EC).

The upstream EC is considered a functional gatekeeper for the downstream hippocampus (Fernandez and Tendolkar, 2006; Basu et al., 2016; Hansen et al., 2018), feeding multi-sensory and associative information into the DG and other hippocampal regions (Bekinschtein et al., 2013; Morrissey and Takehara-Nishiuchi, 2014; Reagh and Yassa, 2014; Kitamura et al., 2015; Save and Sargolini, 2017; Reagh et al., 2018; Morales et al., 2021; Marks et al., 2022). In addition to the EC being involved in spatial navigation and episodic memory, clinical and basic studies suggest that it is also involved in behavioral pattern separation (Yassa et al., 2010; Vivar et al., 2012; Burke et al., 2018). For example, one study suggests that the EC—and particularly the lateral EC (LEC)—and the neighboring perirhinal cortex (PRH) are critical for behavioral pattern separation (Vivar et al., 2012). In the DG-dependent, touchscreen-based location discrimination reversal paradigm (LDR, 16 days probe session), male mice who received LEC/PRH excitotoxic lesions took on average more trials to reach task criterion compared to mice that received control infusions when the load on pattern separation was high (two stimuli were close to each other), but not when the load was low (stimuli were far apart from each other). Thus, the study by Vivar and colleagues shows the importance of LEC/PRH integrity to behavioral pattern separation. While seminal, this lesion study was unable to address two issues. First, as LDR data were averaged over 16 days, it remained unclear how LEC/PRH lesions disrupted behavior over time. Second, the study reported LDR performance before the first reversal—which reflects behavioral pattern separation—but not the performance after the first reversal—which reflects cognitive flexibility (Swan et al., 2014). A more recent study used cell-type-specific ablation to test the role of LEC activity in making associations crucial for behavioral pattern separation (Vandrey et al., 2020). Specifically, cre-mediated ablation of LEC layer IIa (LECIIa) fan cells in male mice impaired complex associative episodic learning (e.g., object-place-context) but not simpler associative learning (Vandrey et al., 2020). LECIIa fan cells are notable as they comprise the only projection from the EC to the DG; they make excitatory synapses in the DG molecular layer (Mol) on the processes of glutamatergic DG granule cells (GCs), adult-born GCs, mossy cells, and GABAergic interneurons (Witter, 2007; Witter et al., 2017; Vandrey et al., 2020; Traub and Whittington, 2022). All of these DG cell types have been implicated in behavioral pattern separation (Leutgeb et al., 2007; Myers and Scharfman, 2009; Jinde et al., 2012; Schmidt et al., 2012; GoodSmith et al., 2017; Nakazawa, 2017; Morales et al., 2021), and some of them—including adult-born DG GCs—have also been implicated in cognitive flexibility (Burghardt et al., 2012; Swan et al., 2014; Yagi and Galea, 2019; Wingert and Sorg, 2021). This circuit positioning of LECIIa fan cells suggests that they are a “load-sensitive” component of the EC-DG circuit in behavioral pattern separation and cognitive flexibility, but this has not yet been tested.

We hypothesized the stimulation of LECIIa fan cells, and thus the stimulation of the LEC → DG projection would improve behavioral pattern separation and cognitive flexibility. To test this, we modified an approach that was previously used to stimulate both LECIIa fan cells and medial EC (MEC) layer IIa stellate cells (Yun et al., 2018). Specifically, we used viral-mediated knockdown via shRNA of tetratricopeptide repeat-containing Rab8b-interacting protein (TRIP8b), a brain-specific auxiliary subunit of the hyperpolarization-activated cyclic nucleotide-gated channel (HCN) (Yun et al., 2018). Relative to control shRNA mice, mice that had EC TRIP8b shRNA in ECIIa fan/stellate cells had several indices of increased EC activity: more firing in EC cells that projected to the DG and hippocampus, higher levels of DG activity-dependent processes (e.g., DG neurogenesis, dendritic arborization), and improved hippocampal-dependent contextual memory. Here, we narrowed our focus to the LEC to fill the knowledge gap on whether behavioral pattern separation and cognitive flexibility are influenced by the activity of LECIIa fan cells that project to the DG (LEC → DG neurons). We report that relative to control mice, mice with TRIP8b shRNA in LECIIa fan cells had improved behavioral pattern separation and cognitive flexibility as well as more DG neurogenesis. Our findings clarify the circuitry engaged in these cognitive abilities, implicating the activity of the “upstream” LEC in behavioral pattern separation and cognitive flexibility.

## Methods and materials

### Animals and ethics statement

The experiments were approved by the Institutional Animal Care and Use Committee at the Children's Hospital of Philadelphia (CHOP) and performed in compliance with the National Institutes of Health Guide for the Care and Use of Laboratory Animals. The mice were group-housed by treatment in an AAALAC-accredited, specific pathogen-free conventional vivarium at the CHOP. Six-week-old C57BL/6J male mice were purchased from Jackson Laboratory (stock number: 000664) and housed in a CHOP vivarium for at least 1 week before the start of the study. Room environments were maintained according to the Guide standards (20–23°C and 30–70% humidity). Home cages consisted of individually ventilated polycarbonate microisolator cages (Lab Products Inc., Enviro-Gard™ III, Seaford, DE) with HEPA-filtered air, corncob (Bed-o' Cobs^®^ ¼”) bedding, provision of one nestlet (Ancare), and a red plastic hut (Bio-Serv, #K3583 Safe Harbor). Each cage of four mice was randomly assigned to the scrambled shRNA (SCR) or TRIP8b shRNA group. Individual mice were identified using the ear punch system. The mice were kept on a 12-h (h) light/dark cycle (lights on at 06:15) with *ad libitum* access to chow (Lab Diets 5015 #0001328) and water. After starting touchscreen experiments, mice were given access to chow from 13:30 to 17:30 (4 h/day [d]), Monday through Thursday, and *ad libitum* access from Friday 13:30 to Sunday 17:30. Mouse weight was recorded at surgery and weekly thereafter to ensure that mice remain above 85% of their initial weight. The data from *n* = 2 SCR shRNA mice were excluded from this experiment, as detailed in the Rigor, Additional ARRIVE 2.0 Details, and Statistical Analysis section below.

### AAV vectors

shRNAs were designed to target the TRIP8b C-terminus region (exon 11) as previously published (Lewis et al., 2009). The following oligonucleotides were used with overhanging ends identical to those created by Sap I and Xba I restriction enzymes: For TRIP8b shRNA, 5′-TTTGAGCATTTGAAGAAGGCTTAATTCAAGAGATTAAGCCTT.

CTTCAAAT GCTATTTTT-3′; SCR shRNA, 5′-TTTGTTCTCCGAACGT.

GTCACGTTTCAAGAGAACGTGACACGTTCGGAGAATTTTT-3′. Hairpin oligonucleotides were phosphorylated by T4 polynucleotide kinase (New England Biolabs, Beverly, MA) followed by annealing at 100°C for 5 min and cooling in the heat block for 3 h. Each annealed oligonucleotide was ligated into the adeno-associated virus (AAV2) plasmid (pAAV-EGFP-shRNA; Stratagene, La Jolla, CA) (Hommel et al., 2003). Virus production was achieved from the UPenn Vector core (Yun et al., 2018).

### Stereotaxic surgery

The mice were anesthetized with a mixture of ketamine (120 mg/kg) and xylazine (16 mg/kg) in saline (0.9% NaCl, i.p.). A bilateral stereotaxic injection of 0.4–0.5 μl of purified high titer AAV (TRIP8b shRNA or SCR shRNA) was directed into the LEC (from Bregma: A/P-3.7 mm, M/L+4.4, angle 4°; from Lambda: D/V-4.5) using 33 gauge Hamilton syringes (Hamilton, Reno, NV). The injection rate was 0.1 μl/min, with needles kept in place for an additional 5 min to enable diffusion.

### Overview of behavioral testing

**Figure 1:**
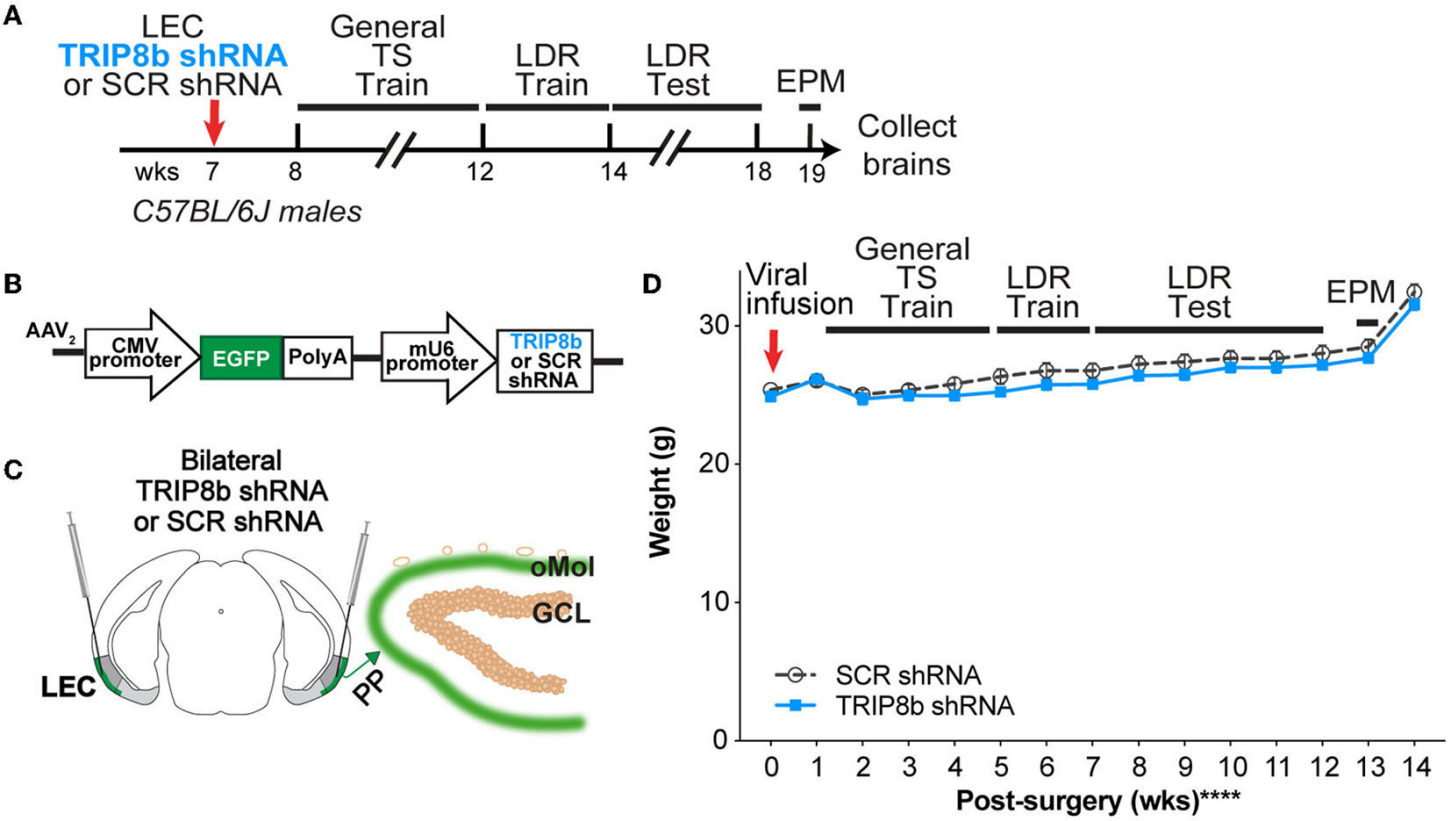
Timeline and overview of the experiment. **(A)** Timeline for the experiment. Seven-week-old C57BL/6J male mice received bilateral infusions (red arrow) of AAV-TRIP8b short-hairpin (TRIP8b shRNA) or scrambled shRNA (SCR shRNA) into the lateral entorhinal cortex (LEC) to knockdown (KD) the HCN auxiliary protein TRIP8b as done in prior work. After sufficient time for the virus to express encoded proteins, mice went through touchscreen training (General TS Train) with a 12-window grid (2X6) followed by location discrimination reversal training (LDR Train) and testing (LDR Test). After the touchscreen experiments were finished, mice were run on the elevated plus maze (EPM) to gauge innate fear of open vs. closed EPM arms, and then brains were collected. **(B)** Schematic of TRIP8b shRNA construct packaged into adeno-associated virus (AAV_2_) also expresses EGFP. For AAV-expressing SCR shRNA, the construct was the same with the exception that SCR shRNA (not the TRIP8b shRNA) was under the control of the mU6 promoter. [**(C)** left] Schematic of a coronal section through an adult mouse brain depicting bilateral LEC AAV infusions (indicated by syringes) given via stereotaxic surgery. Mice received a LEC-directed virus containing either TRIP8b shRNA or control scrambled (SCR) virus. **[(C)** right**]** Schematic of a portion of an enlarged coronal section through the hippocampal dentate gyrus (DG) ~4 weeks post-surgery. When processed for immunohistochemistry, “on target” LEC viral infusions would result in the expression of fluorescence protein (EGFP+, green) in the LEC projections that course through the perforant path (PP) and terminate in the DG outer molecular layer (oMol, green). **(D)** Body weight in TRIP8b shRNA vs. SCR shRNA mice throughout the experiment shown in **(A)**. Mean+/-SEM. Two-way RM ANOVA was performed in **(D)**. Main effects: Time *F* (14, 238) = 130.6, ****p < 0.0001 and Treatment *F* (1, 17) = 0.6704, *p* = 0.4242; Interaction: Treatment X Time *F* (14, 238) = 0.9406, *p* = 0.5158. CMV, cytomegalovirus; GCL, granule cell layer; wks, weeks. Complete statistical information is provided in Supplementary Table 1.

The mice with an infusion of SCR shRNA or TRIP8b shRNA virus in the LEC (Figure 1A) underwent touchscreen behavioral testing 1-week post-surgery. Touchscreen experiments were performed between 08:00 and 14:00 on weekdays (Soler et al., 2021). As is standard in most rodent touchscreen experiments, the mice were food restricted during touchscreen experiments. Mouse chow was removed from each cage at 5 pm the day before training or testing. Each cage was given *ad libitum* access to chow for 3 h (minimum) to 4 h (maximum) immediately following daily touchscreen training/testing, and from completion of training/testing on Friday until Sunday 5 p.m. The mice were weighed weekly to ensure weights were >85% of initial body weight. While weights below this threshold merited the removal of the mouse from the study, zero mice reached this threshold (Mar et al., 2013; Oomen et al., 2013). Operant touchscreen platform procedures included general touchscreen training (with 2 x 6 window grid) and location discrimination reversal (LDR) training (LDR Train) and testing (LDR Test); LDR was selected for its ability to reflect an animal's performance on both behavioral pattern separation and cognitive flexibility (Swan et al., 2014). After all the mice completed LDR testing, they received unrestricted food pellets for 1 week before behavior testing on the elevated plus maze (EPM) for innate fear/anxiety measurement. The subject number overview is provided in the Statistical Analysis section below, and the subject number for each group in each figure panel is provided in Supplementary Table 1.

General Touchscreen Training (before LDR), or “General TS Train” (Figure 1A), consists of five stages (Whoolery et al., 2020; Soler et al., 2021): Habituation, Initial Touch, Must Touch, Must Initiate, and Punish Incorrect (PI). Methods for each stage are described in turn below. Mice went through General TS Train with 12 windows (2 X 6) for the LDR experiment.

#### Habituation

Mice are individually placed in a touchscreen chamber for 30 min (maximum) with the magazine light turned on (LED Light, 75.2 lux). For the initial reward in each Habituation session, a tone is played (70 decibels [dB] at 500 Hz, 1,000 ms) at the same time as a priming reward (150 ul Ensure^®^ Original Strawberry Nutrition Shake) is dispensed to the reward magazine. After a mouse inserts and removes its head from the magazine, the magazine light turns off and a 10-s (s) delay begins. At the end of the delay, the magazine light is turned on and the tone is played again as a standard amount of the reward (7 ul Ensure) is dispensed. If the mouse's head remains in the magazine at the end of the 10 s delay, an additional 1 s delay is added. A mouse completes Habituation training after they collect 25 rewards (25 x 7 ul) within 30 min. The mice that achieve Habituation criteria in <30 min are removed from the chamber immediately after their 25th reward to minimize extinction learning. The measure reported for Habituation is days to completion.

#### Initial touch

A 2 x 6 window grid is placed in front of the touchscreen for the remaining stages of training. At the start of the session, an image (a lit white square) appears in a pseudorandom location in one of the 12 windows on the touchscreen. The mouse has 30 s to touch the lit square (typically with their nose). If the mouse does not touch the image, it is removed, a reward (7 ul Ensure) is delivered into the illuminated magazine on the opposite wall from the touchscreen, and a tone is played. After the reward is collected, the magazine light turns off and a 20-s intertrial interval (ITI) begins. If the mouse touches the image while it is displayed, the image is removed and the mouse receives three times the normal reward (21 ul Ensure, the magazine is illuminated, and the tone is played). For subsequent trials, the image appears in another of the 12 windows on the touchscreen, and never in the same location more than three consecutive times. The mice reach the criteria and advance past Initial Touch training when they complete 25 trials (irrespective of reward level received) within 30 min. The mice that achieve Initial Touch criteria in <30 min are removed from the chamber immediately after their 25th trial. The measure reported for Initial Touch is days to completion.

#### Must touch

Similar to Initial Touch training, an image appears, but now the window remains lit until it is touched. If the mouse touches the lit square, the mouse receives a reward (7 ul Ensure, the magazine is illuminated, and the tone is played). If the mouse touches one of the blank windows, there is no response (no reward is dispensed, the magazine is not illuminated, and no tone is played). The mice reach the criteria and advance past Must Touch training after they complete 25 trials within 30 min. Mice that achieve Must Touch criteria in <30 min are removed from the chamber immediately after their 25th trial. The measure reported for Must Touch is days to completion.

#### Must initiate

Must Initiate training is similar to Must Touch training, but a mouse is now required to initiate the training by placing its head into the already illuminated magazine. A random placement of the image (lit white square) will then appear on the screen, and the mouse must touch the image to receive a reward (7 ul Ensure, magazine lit, and tone played). Following the collection of the reward, the mouse must remove its head from the magazine and then reinsert its head to initiate the next trial. The mice advance past the Must Initiate training after they complete 25 trials within 30 min. The mice that achieve Must Initiate criteria in <30 min are removed from the chamber immediately after their 25th trial. The measure reported for Must Initiate is days to completion.

#### Punish incorrect (PI)

Punish Incorrect training builds on the Must Initiate training, but here if a mouse touches a portion of the screen that is blank (does not have a lit white square), the overhead house light turns on and the lit white square disappears from the screen. After a 5-s timeout period, the house light turns off and the mouse has to initiate a correction trial where the lit white square appears in the same location on the screen. The correction trials are repeated until the mouse successfully presses the lit white square; however, correction trials are not counted toward the final percent correct criteria. The mice reach the criteria and advance past the Punish Incorrect training and onto the LDR Train/Test after they complete 30 trials within 30 min at ≥76% (≥19 correct) on Day 1 and >80% (>24 correct) on Day 2 over 2 consecutive days. The mice that achieve PI criteria in <30 min are removed from the chamber immediately after their 30th trial. As with the other stages, a measure reported for PI is days to completion (to reach criteria). However, since the PI stage also contains a metric of accuracy, more measures were analyzed relative to the other five stages. Therefore, other measures reported for PI are session length, trial number, and percent correct responses.

Location Discrimination Reversal (LDR; program LD1 choice reversal v3; ABET II software, Cat #89546-6) tests the ability to discriminate two conditioned stimuli that are separated either by a Large or Small distance or separation. The reversal component of LDR is used here and, as in classic LDR studies (Clelland et al., 2009; Oomen et al., 2013; Swan et al., 2014; Soler et al., 2021), is used to test cognitive flexibility. Taken together, LDR is a hippocampal-dependent task that allows assessment of both discrimination ability as well as cognitive flexibility. In our timeline (Figure 1A), mice received one additional training step (“LDR Train”) before the actual two-choice LDR Test.

#### Location discrimination reversal train (LDR Train)

The mice initiate the trial, which leads to the display of two identical white squares (25 x 25 pixels, Figure 3A) presented with two blank (unlit) squares between them, a separation which is termed “intermediate” (8th and 11th windows in 2 x 6 high grid-bottom row). One of the left or right locations of the squares is rewarded (i.e., S+) and the other is not (S-), and the initial rewarded location (left or right) is counterbalanced within-group. On subsequent days, the rewarded square location is switched based on the previous day's performance. The reward side is carried over from the previous session/day if they did not reach the criteria (+1 reversal). A daily LDR Train session is complete once the mouse runs 50 trials or when 30 min has passed. Once seven out of eight trials had been correctly responded to, on a rolling basis, the rewarded square location was switched (becomes S-), then S+, then S-, etc.; this is termed a “reversal.” Once the mouse reaches >1 reversal in three out of four consecutive testing sessions, the mouse advances to the LDR Test. Measures reported for LDR Train are the percent of subjects to reach completion, days to completion, percent correct for all trials in a given day (Day 1 vs. Last Day), time to first reversal (Day 1 vs. Last Day), and percent correct to first reversal (Day 1 vs. Last Day).

#### Location discrimination reversal test (LDR Test)

The mice initiate the trial, which leads to the display of two identical white squares, either with four black squares between them (“Large” separation, two at maximum separation [7th and 12th windows in the bottom row of a 2 x 6 grid]) or directly next to each other (“Small” separation, two at minimum separation [9th and 10th windows in the bottom row of a 2 x 6 grid; Figures 4A, B]). As in the LDR Train, only one of the square locations (right-most or left-most) is rewarded (S+, same side for both Large and Small separation, and counterbalanced within groups). The rewarded square location is reversed based on the previous day's performance (S+ becomes S-, then S+, then S-, etc.). Once seven out of eight trials correctly responded to, on a rolling basis, the rewarded square location is reversed (becomes S-, then S+, then S-, etc.). A daily LDR Test session is complete once the mouse touches either S+ or S- 81 times or when 30 min has passed. Each mouse is exposed to only one separation type during a daily LDR Test session (either Large or Small) and the separation type changes every 2 days (2 days of Large, then 2 days of Small, counterbalanced across mice). The mice are exposed to six “blocks” of the LDR Test, where 1 block = 4 days LDR Test counterbalanced with two Large and two Small separation daily sessions. Once 24 test days (12 days of Large, 12 days of Small separation, a total of six blocks) are complete, mice receive a 1-week normal feeding before subsequent behavior testing and brain collection. Reported are metrics of LDR Test performance *before* the first reversal—session length and percent correct during trials to (or before) the first reversal—which reflect behavioral pattern separation (Swan et al., 2014). Also reported is a metric of LDR Test performance *after* the first reversal—number of reversals—which reflects cognitive flexibility (Swan et al., 2014). These metrics relevant to behavioral pattern separation and cognitive flexibility are presented from the last day of Large and Small separation from both Block 1 and Block 6. Additional LDR Test measures reported reflecting attention (correct image choice latency), motivation (reward collection latency), and impulsivity (total ITI blank touches), which are also presented for both Large and Small separation.

#### Elevated plus maze (EPM)

The EPM consists of two open arms (L 67 x W 6 cm) and two closed arms with walls (L 67 x W 6 x H 17 cm, opaque gray Plexiglas walls and black Plexiglas floor, Harvard Apparatus, #760075). At the start of the test, the mice are placed on the far end of the open arms and allowed free movement throughout the maze for 5 min. The parameters of the EPM (total distance moved, number of entries, and duration spent in the open and closed arms) were scored via EthoVisionXT software (Noldus Information Technology) using nose-center-tail tracking to determine position.

### Duration of experiment

During the five stages of the General TS Train, all SCR shRNA and TRIP8b shRNA mice gained touchscreen-related operant learning experience, which was followed by the LDR Train and LDR Test. After completing the LDR Test, all mice underwent EPM to gauge innate fear of open vs. closed EPM arms. Brains were collected after the EPM test. Thus, the duration of the experiment (from the beginning of General TS Train to brain collection) was 3 months.

### Brain collection and brain section preparation

A single cage of mice was brought into the procedure room at a time, and all mice in the cage were decapitated using IACUC-approved scissors within 3 min. The brains were immersion fixed with 4% paraformaldehyde in 1XPBS at room temperature for 3 d followed by cryoprotection (placement in 30% sucrose in 1XPBS at room temperature for 3 more days and then stored at 4°C until sectioning). The brains were sectioned coronally on a microtome (Leica) by covering the brain with fine dry ice and collecting 30 μm sections through the entire anterior-posterior length of the hippocampus and entorhinal cortex (distance range from Bregma: −0.82 to −4.24 μm). Serial sets of sections were stored in 0.1% NaN_3_ in 1XPBS at 4°C until processing for slide-mounted immunohistochemistry (IHC) (Ables et al., 2010; Lagace et al., 2010).

### Immunohistochemistry (IHC)

For single-labeling of tissue with antibodies against either doublecortin (DCX) or GFP, one series of sections was mounted on glass slides (Superfrost/Plus, Fisher) and coded to ensure the experimenters remained blind throughout quantification and data analysis. Sections were processed for antigen retrieval (0.01 M citric acid, pH 6.0, 95°C, 15 min) and non-specific staining was blocked by incubating in blocking solution (3% normal donkey serum [NDS], vol/vol in 0.1% Triton X-100 in 1 XPBS) for 30 min. After blocking, sections were then incubated in either goat anti-DCX (1:5,000; Santa Cruz, Cat. #SC-8066) or chicken anti-GFP (1:3,000; Aves Cat. #GFP-1020) in 0.1% Tween-20 in 1XPBS overnight. The following day, sections were rinsed and incubated in either biotinylated-donkey anti-goat IgG antibody (Cat. #705-065-003) or biotinylated-donkey-α-chicken-IgY (Cat. #703-065-155), both 1:200 (Jackson ImmunoResearch Laboratories Inc., West Grove, PA) in 1.5% NDS in 1XPBS for 1 h. After rinsing in 1 XPBS and 30 min in 0.3% hydrogen peroxide in 1 XPBS to quench endogenous peroxidases, sections were incubated in avidin-biotin complex (ABC Elite, Vector Laboratories) for 60 min. After rinsing in 1XPBS, staining for DCX immunoreactive (+) cells was visualized using DAB (Thermo Scientific, Cat. #1856090), and for GFP+ cells was visualized using Fluorescein-labeled Tyramide (PerkinElmer, Cat. #SAT701). Nuclear Fast Red (Vector Laboratories, Cat. #H-3403) or DAPI (Roche, Cat. #236276) were used as counterstains for DCX and GFP immunolabeling, respectively.

### Targeting LEC → DG neurons

Accurate virus targeting was verified after brain collection. Specifically, TRIP8b shRNA mice with GFP+ soma in LEC II/III, GFP+ processes in the perforant path, GFP+ terminals in the middle and/or outer DG Mol—but with no GFP+ projections in other hippocampal regions, such as CA1—were considered “on target.” TRIP8b shRNA mice that also had fine projections in non-DG hippocampal regions, such as in the stratum lacunosum-moleculare (SLM), were considered to have too broad of viral expression to be considered on target. Images and functional importance of this targeting are provided in Supplementary Figure 1. No SCR shRNA mice were excluded based on GFP+ processes and terminals since this construct is considered to be biologically inactive.

### Quantification of DCX-immunoreactive (DCX+) ± cells

Unbiased quantification of DCX+ cells (total as well as late progenitors and immature neurons in the superior [or suprapyramidal] and inferior [or infrapyramidal] blade of the DG) was performed via stereology (Latchney et al., 2014; Whoolery et al., 2017; Luna et al., 2019; Clark et al., 2021). The microscope used was a BX51 (Olympus America, Center Valley, PA, USA) with a 40X, 0.63 NA oil-immersion objective. DCX+ cells in the DG subgranular zone (SGZ) were counted. The total DCX+ cell number was calculated by this formula (Yun et al., 2018; Clark et al., 2021):


$$\begin{array}{l} \text{Total population of cells}=\text{total cells counted}\times 1\text{/}\text{ssf}\\ \;\times 1\text{/}\text{asf}\times 1\text{/}\text{hsf} \end{array}$$

where ssf is the section sampling fraction (DCX: 1/8), asf is the area sampling fraction (one for these rare populations of cells; thus, all cells were counted in “ssf [e.g., 1/8]” sections), and hsf is the height sampling fraction (one given the minimal effect edge artifacts have in counting soma <10 um with ssf 1/8) as described in prior work (Lagace et al., 2010). One hemisphere in hippocampal dorsal DG (spanning approximately−0.95 mm to −2.65 mm from Bregma) was counted for DCX+ cells, thus the resulting formula was:


$$\begin{array}{l} \text{Total population of DCX}+\text{cells}=[\text{total cells counted}\times 1/(1/8)\\ \;\times 1/1\times 1/1]\times 2 \end{array}$$

Therefore, the DCX+ cell counts were multiplied by 16 to obtain the total number of DCX+ GC layer (GCL) cells. Similar formulas were applied for DCX+ subtypes (progenitors and immature neurons) which were classified based on their soma morphology and absence/presence of processes.

### Computer scripts

Before statistical analysis, the touchscreen data were sorted and extracted. We used a custom Python 3.8.6 and Pycharm script developed by the Eisch Lab to extract to calculate the needed values and compile the data into a database. Extracting the data into an output CSV file was managed with another custom script, and these outputs were verified manually. Following this verification, the data were analyzed using GraphPad Prism 9 according to the tests detailed in the Statistical Analysis section. These scripts along with sample data files are available at https://github.com/EischLab/ts-data-analysis-app.

### Rigor, additional ARRIVE 2.0 details, and statistical analysis

The experimental unit in this study is a single mouse. For behavioral studies, the mice were randomly assigned to groups. Steps were taken at each experimental stage to minimize potential confounds. For example, the mice from the two experimental groups (SCR shRNA and TRIP8b shRNA) were interspersed throughout housing racks at CHOP (to prevent the effects of cage location). Sample sizes were pre-determined via power analysis and confirmed based on extensive laboratory experience and consultation with CHOP and PennMed statisticians, as previously reported (Whoolery et al., 2020; Soler et al., 2021). The exact subject number for each group is provided in Supplementary Table 1. A total of *n* = 2 SCR shRNA mice were outliers based on *a priori* established experimental reasons (*n* = 1 SCR shRNA mice did not complete the PI stage even by Day 33; *n* = 1 Sham did not complete LDR “Acquisition” since it reached a humane endpoint), and the data from these mice were excluded from this experiment. The initial subject numbers per group were SCR shRNA (*n*) = 11 and TRIP8b shRNA (*n*) = 16. After assessment for GFP+ terminals restricted to the DG Mol, the subject numbers per group were SCR shRNA (*n*) = 9 and TRIP8b shRNA (*n*) = 10, and these were the mice whose data are presented in the main figures. The experimenters were blinded to treatment until the analysis was complete. Data for each group are reported as mean ± s.e.m (line graphs) or median and quartile (truncated violin plots, solid lines indicating quartile, and dotted lines indicating median). Testing of data assumptions (normal distribution, similar variation between control and experimental groups, etc.) and statistical analyses were performed in GraphPad Prism. D-Agostino's tests and the QQ plot were chosen for normality tests. Since the data in all figures were normally distributed, parametric tests were used for analysis. Statistical approaches and results including statistical analysis significance (*p*-values) and effect size (when RM two-way ANOVA or one-way ANOVA, *p* < 0.05: partial omega-squared ωp2 where ≤ 0.05 small, ≥0.06 medium, and ≥0.14 large) are provided in the Results section and/or Supplementary Table 1. Analyses were hypothesis-based and therefore pre-planned unless otherwise noted in the Results section. Our hypothesis is based on treatment effects (SCR shRNA vs. TRIP8b shRNA mice), and results that showed a treatment effect or interaction were considered the most relevant to testing this hypothesis. Therefore, in general, such results are noted in the Abstract section and discussed in the Discussion section. Other main effects (training stages, Large vs. Small separation, or Day 1 vs. Last Day) are fully presented in the Results section but not emphasized in the Abstract or Discussion section. Analyses with two groups were performed using an unpaired, two-tailed Student's *t*-test. Analyses with more than two variables were performed using a two-way ANOVA or a mixed-effects analysis with Bonferroni *post hoc* test; repeated measures (RM) were used where appropriate, as indicated in Figure Legends and Supplementary Table 1. Analysis of the distribution of subjects reaching criteria between control and experimental groups (survival curve) was performed with the Mantel–Cox test. While significance was defined as ^*^p < 0.05, the effect size was also considered when evaluating statistical analyses (see Supplementary Table 1).

## Results

### In C57BL/6J male mice, LEC SCR shRNA mice and TRIP8b shRNA mice have similar weight gain and similar performance in operant training on a touchscreen platform

C57BL/6J male mice received bilateral LEC infusions of either SCR shRNA or TRIP8b shRNA 1 week before the start of the touchscreen experiments (Figures 1A, B). This results in GFP+ LEC stellate cells in layers II/III (LECII/III) and GFP+ processes in the perforant path and DG Mol (Figure 1C) (Yun et al., 2018); mice with GFP+ processes in non-DG regions of the hippocampus were excluded from most of the data shown in the main text (see below and Supplementary Figure 1). While our viral construct can be expressed in non-stellate LEC cells, TRIP8b in the LEC is primarily expressed in stellate cells (Wilkars et al., 2012). Thus, we consider this targeting to knockdown TRIP8b in LECII/III stellate cells and their afferents to the DG Mol, which enhances DG activity-dependent processes, such as neurogenesis and c-fos expression, and improves simple hippocampal-dependent learning and leads to behavior that could be considered “antidepressive-like” (Yun et al., 2018).

**Figure 2:**
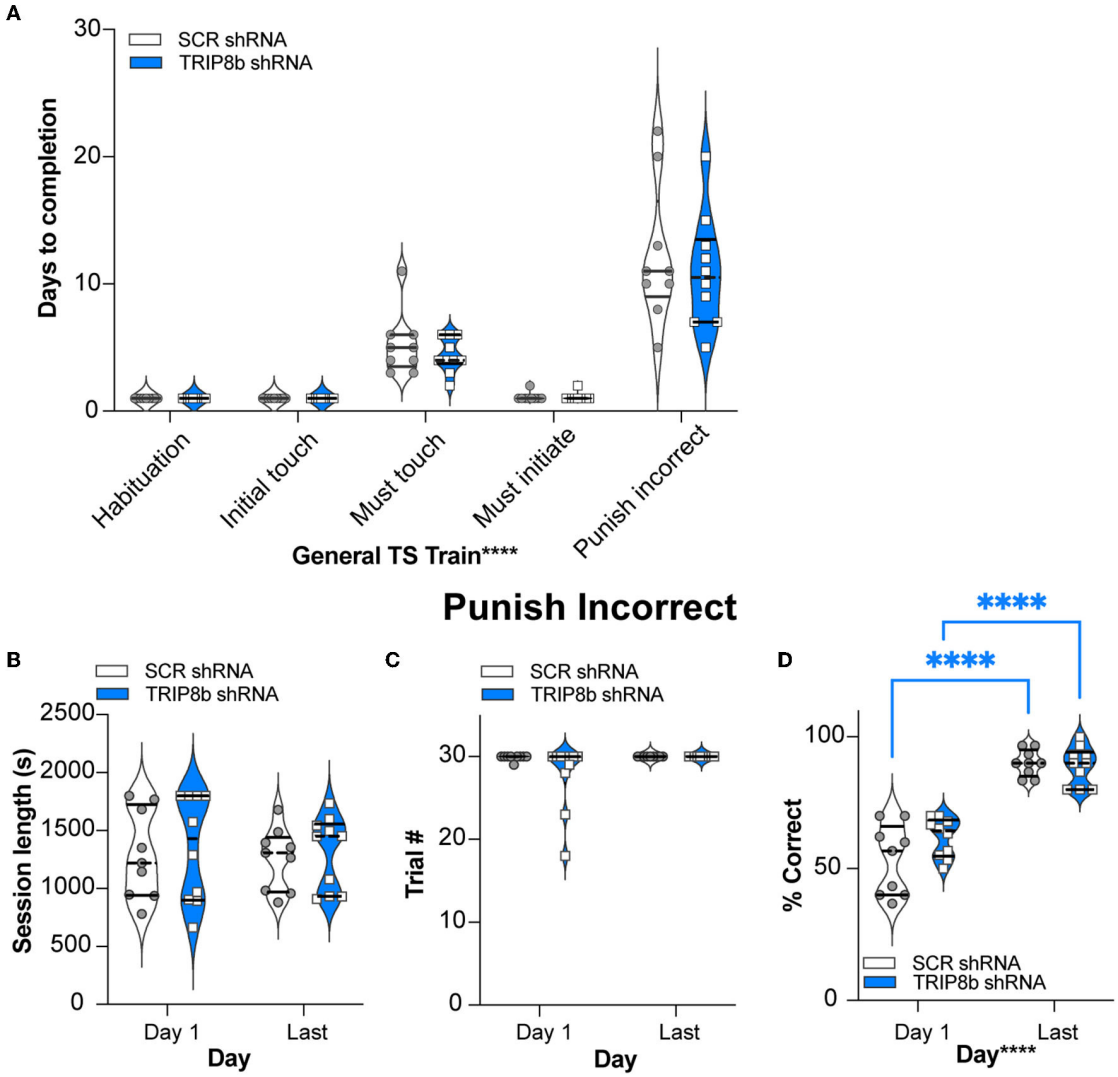
Knockdown of TRIP8b in the LEC does not change performance in general TS Train. **(A)** In the five stages of General TS Train (Habituation, Initial Touch, Must Touch, Must Initiate, and Punish Incorrect), SCR shRNA and TRIP8b shRNA mice took a similar number of days to reach the criteria. **(B–D)** In other criteria (beyond Days) for the Punish Incorrect stage of General TS Train, SCR shRNA and TRIP8b shRNA mice also performed similarly on both Day 1 and the Last Day: session length **(B)**, number of trials **(C)**, and % correct (accuracy). **(D)** Note both SCR and TRIP8b mice showed increased accuracy between the first and last day of Punish Incorrect. Two-way RM ANOVA was performed in **(A–D)**: **(A)** Main effects: Training Stage *F* (4, 68) = 75.03, ****p < 0.0001 and Treatment *F* (1, 17) = 0.6292, *p* = 0.4386; Interaction: Training Stage x Treatment *F* (4, 68) = 0.3333, *p* = 0.8547. **(B)** Main effects: Training Stage *F* (1, 17) = 0.08579, *p* = 0.7731 and Treatment *F* (1, 17) = 0.2492, *p* = 0.6240; Interaction: Training Stage x Treatment *F* (1, 17) = 2.128e−005, *p* = 0.9964. **(C)** Main effects: Training Stage *F* (1, 17) = 2.858, *p* = 0.1092 and Treatment *F* (1, 17) = 2.335, *p* = 0.1449; Interaction: Training Stage x Treatment *F* (1, 17) = 2.335, *p* = 0.1449. **(D)** Main Effects: Training Stage *F* (1, 17) = 148.2, ****p < 0.0001 and Treatment *F* (1, 17) = 1.599, *p* = 0.2231, *post hoc*: ****p < 0.0001 in Day 1 vs. Last Day in both SCR and TRIP8b; Interaction: Training Stage x Treatment *F* (1, 17) = 3.358, *p* = 0.0844. LEC, lateral entorhinal cortex; s, seconds; S+, stimulus associated with a reward; TS, touchscreen. In these truncated violin plots, solid lines indicate quartile and dotted lines indicate median values. Complete statistical information is provided in Supplementary Table 1.

The SCR shRNA and TRIP8b shRNA mice had similar weight gain throughout the experiment (Figure 1D, Supplementary Table 1), consistent with prior work (Yun et al., 2018). In addition, the SCR shRNA and TRIP8b shRNA mice completed each stage of the general touchscreen training in similar periods (Figure 2A, Supplementary Table 1). During the PI stage, both SCR shRNA and TRIP8b shRNA mice showed high variability in days to completion. Therefore, we examined additional PI parameters on Day 1 vs. Last Day. The SCR shRNA and TRIP8b shRNA mice had similar PI session lengths (Figure 2B, Supplementary Table 1) and a number of trials (Figure 2C, Supplementary Table 1). However, there was a main effect of day on Percent Correct. A post-hoc analysis of Day 1 and Last Day showed both groups had higher accuracy on their Last Day vs. their Day 1 (Figure 2D, Supplementary Table 1). Thus, SCR shRNA and TRIP8b shRNA mice performed similarly in the fundamental operant learning that is required on this operant touchscreen platform, with both groups improving accuracy across the duration of the PI training stage.

### There is no effect of treatment (SCR shRNA vs. TRIP8b shRNA) on LDR Train performance

**Figure 3:**
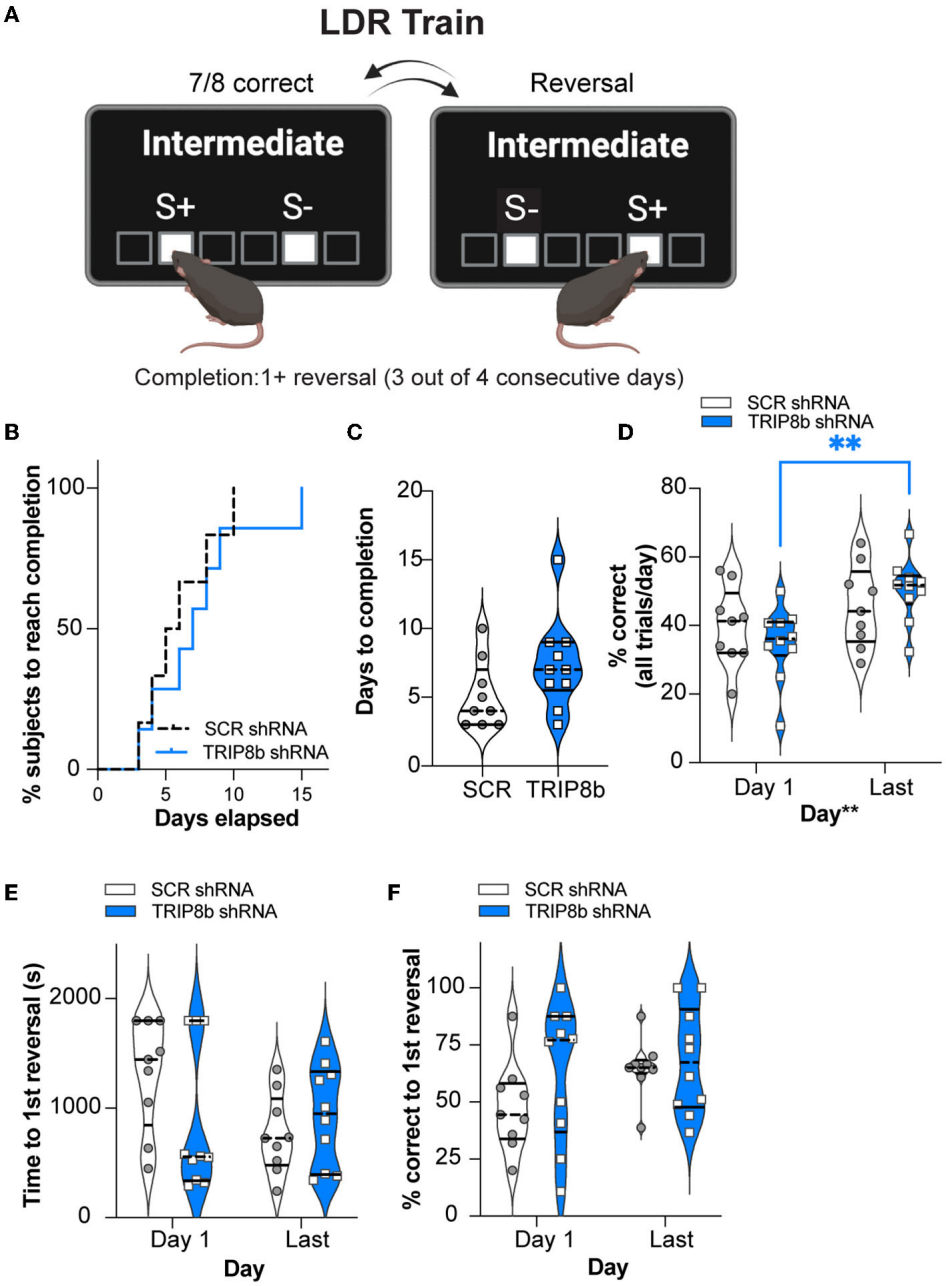
Knockdown of TRIP8b in the LEC improved accuracy between the Last Day and Day 1 of the LDR Train. **(A)** Schematic depicting LDR Train sessions, criteria for reversal, and criteria for LDR Train completion. When mice made seven out of eight consecutive correct choices of stimulus (S+), a reversal occurred (S+ became S- [incorrect stimulus] and the previous S- became S+). Criteria for LDR Train completion is a mouse making at least 1 (1+) reversal on 3 out of 4 consecutive days. **(B, C)** LDR Train data from SCR shRNA and TRIP8b shRNA mice showing **(B)** % subjects that complete LDR Train over days elapsed and **(C)** the total days it took each group to complete the LDR Train. **(D)** There was no effect of treatment on accuracy (% correct), and therefore we consider accuracy over time to be similar in these groups. However, there was a main effect of days, and *post-hoc* analysis showed that accuracy was improved for TRIP8b shRNA mice on the LDR Train on the Last Day vs. Day 1. There was no such improvement in SCR shRNA mice. **(E, F)** On LDR Train Day 1, SCR shRNA and TRIP8b shRNA mice took the same amount of time to reach the first reversal **(E)** and had similar accuracy **(F)**. On the Last Day, there was no difference in speed to first reversal or accuracy between the groups. The Mantel–Cox test in **(B)**, *p* = 0.5328, unpaired *t*-test in **(C)**, *p* = 0.1088, and two-way RM ANOVA in **(D–F)** were performed. **(D)** Main effects: Time *F* (1, 17) = 10.08, **p = 0.0055 and Treatment *F* (1, 17) = 0.0006715, *p* = 0.9796, *post-hoc*: **p = 0.0075 on Day 1 vs. Last Day in TRIP8b; Interaction: Treatment x Time *F* (1, 17) = 2.084, *p* = 0.1671. **(E)** Main effects: Time *F* (1, 17) = 1.386, *p* = 0.2552 and Treatment *F* (1, 17) = 1.500, *p* = 0.2373; interaction: Treatment x Time *F* (1, 17) = 2.382, *p* = 0.1411. **(F)** Main effects: Time *F* (1, 17) = 1.571, *p* = 0.2271 and Treatment *F* (1, 17) = 2.700, *p* = 0.1187; Interaction: Treatment x Time *F* (1, 17) = 0.5309, *p* = 0.4762. LDR, location discrimination reversal; s, seconds. Complete statistical information is provided in Supplementary Table 1.

Having established similar operant learning in the SCR mRNA and TRIP8b shRNA mice, we next assessed their performance in the LDR Train, the precursor to the LDR Test, where lit squares are separated by an intermediate number of unlit squares (Figure 3A). There was a visual difference in the percent of SCR shRNA and TRIP8b shRNA subjects reaching the criteria in LDR Train (perhaps due to one mouse—which was not a statistical outlier—in the TRIP8b group taking 15 days). However, this difference was rejected by survival curve analysis (Figure 3B, Supplementary Table 1). The SCR shRNA and TRIP8b shRNA mice also had similar average days to complete the LDR Train (Figure 3C, Supplementary Table 1). There was no treatment effect on overall accuracy (percent correct for all trials in a given day). There was a main effect of day, however, with *post hoc* analysis showing that TRIP8b shRNA mice had higher accuracy on the Last Day of the LDR Train vs. Day 1 (Figure 3D, Supplementary Table 1). The SCR shRNA and TRIP8b shRNA mice also did not differ in total session time to reach the first reversal (Figure 3E, Supplementary Table 1) or percent correct to the first reversal (Figure 3F, Supplementary Table 1). Thus, in the training step for LDR, there was no treatment effect on performance.

### LEC TRIP8b shRNA mice have improved behavioral pattern separation and cognitive flexibility compared to SCR shRNA mice

After the mice reached the LDR Train criteria, the mice began the LDR Test to assess behavioral pattern separation (performance *before* the first reversal) and cognitive flexibility (performance *after* the first reversal) (Mar et al., 2013; Oomen et al., 2013; Swan et al., 2014; Phillips et al., 2018).

**Figure 4:**
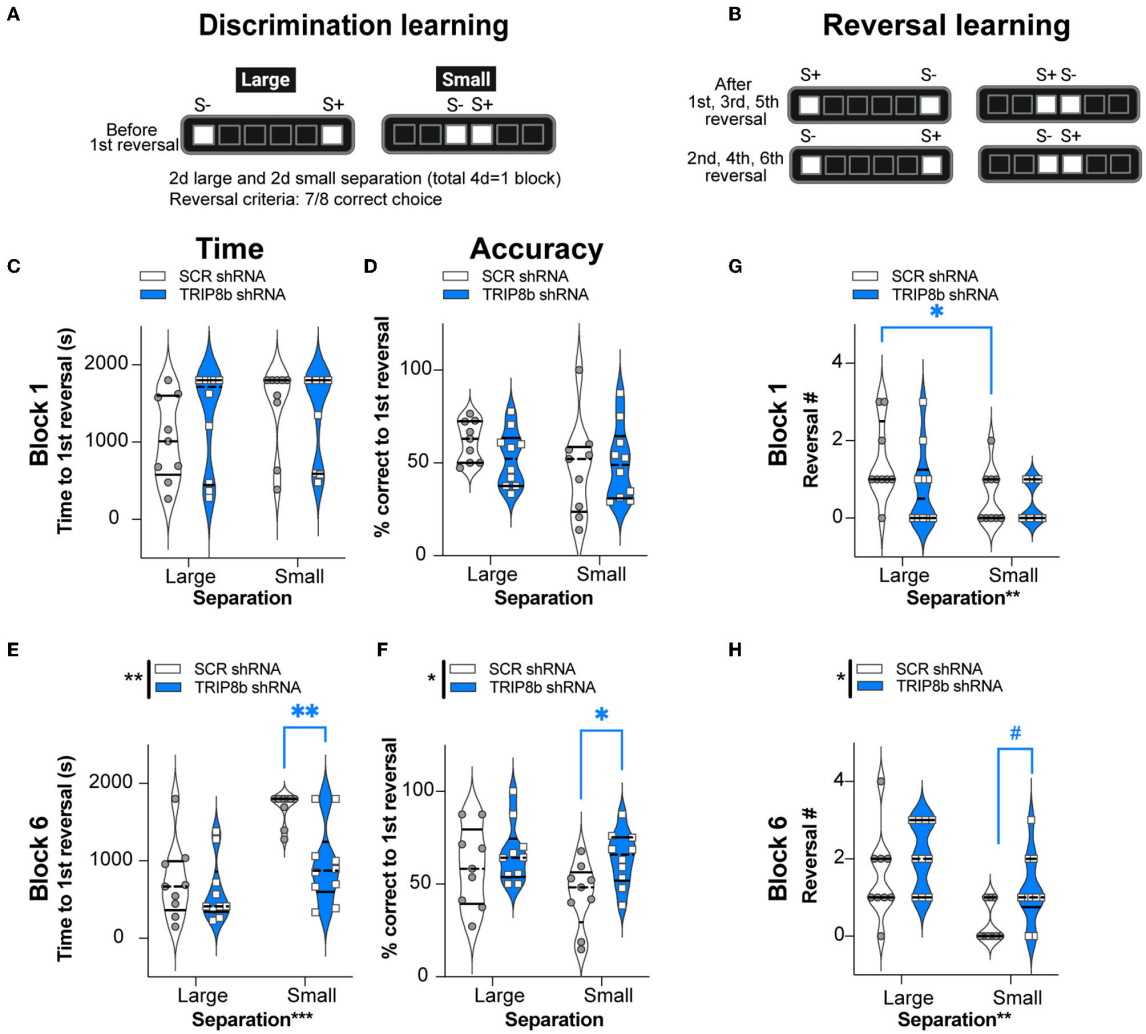
Knockdown of TRIP8b in the LEC improved discrimination learning in the LDR Test. **(A, B)** Schematic depicting LDR Test sessions. The degree of difficulty (or “load”) is provided by the lit square (stimuli, S) separated by either four black squares (Large separation) or zero spaces (Small separation). **(A)** Discrimination learning was tested in the sessions that occurred before the first reversal each day and reflected behavioral pattern separation ability. After selecting the correct choice (S+) in seven out of eight consecutive trials, S+ became S- and the reversal learning trials **(B)** began, which reflects cognitive flexibility. Trials continued with S+ and S- designated as depicted. LDR Test was run for six blocks, where one block consisted of 2 days of Large separation and 2 days of Small separation. **(C–F)** Discrimination Learning. **(C, D)** TRIP8b shRNA and SCR shRNA mice performed with similar speed and accuracy before the first reversal under both Large and Small separation on the Last Day of Block 1. **(E, F)** On the last block (Block 6), there was a treatment effect: TRIP8b mice reached the first reversal faster and with higher accuracy than SCR shRNA mice, specifically under the condition of Small separation. **(G, H)** Reversal Learning. TRIP8b shRNA and SCR shRNA mice made a similar number of reversals in Block 1. However, there was a main effect of Treatment and Separation in Block 6. *Post-hoc* analysis showed no significant difference between groups in Large separation, but a trend toward more reversals (medium effect size, see Supplementary Table 1) in TRIP8b shRNA vs. SCR shRNA mice in Small separation. Two-way RM ANOVA was performed in **(C–F)**. **(C)** Main effects: Separation *F* (1, 17) = 1.084, *p* = 0.3124 and Treatment *F* (1, 17) = 2.423, *p* = 0.1380; interaction: Separation x Treatment *F* (1, 17) = 0.1799, *p* = 0.6768. **(D)** Main effects: Separation *F* (1, 17) = 1.679, *p* = 0.2123 and Treatment *F* (1, 17) = 0.3233, *p* = 0.5771; interaction: Separation x Treatment *F* (1, 17) = 0.8583, *p* = 0.3672. **(E)** Main effects: Separation *F* (1, 17) = 16.36, ***p = 0.0008 and Treatment *F* (1, 17) = 15.53, **p = 0.0011, *post-hoc*: **p = 0.0012 in SCR vs. TRIP8b on Small separation; interaction: Separation x Treatment *F* (1, 17) = 3.589, *p* = 0.0753. **(F)** Main effects: Separation *F* (1, 17) = 1.760, *p* = 0.2022 and Treatment *F* (1, 17) = 8.939, **p = 0.0082*, post hoc*: *p = 0.0397 in SCR vs TRIP8b on Small Separation; interaction: Separation x Treatment *F* (1, 17) = 0.8945, *p* = 0.3575. **(G)** Main effects: Separation *F* (1, 17) = 10.07, **p = 0.0056 and Treatment *F* (1, 17) = 1.448, *p* = 0.2454; interaction: Separation x Treatment *F* (1, 17) = 1.448, *p* = 0.2453, *post hoc*: *p = 0.0156 in Large vs. Small separation of SCR shRNA. **(H)** Main effects: Separation *F* (1, 17) = 14.83, **p = 0.0013 and Treatment *F* (1, 17) = 5.531, *p = 0.0310, *post-hoc*: #*p* = 0.081 in SCR vs. TRIP8b in Small separation; interaction: Separation x Treatment *F* (1, 17) = 0.3419, *p* = 0.5664. LDR, location discrimination reversal; s, seconds, S+=stimulus associated with a reward. Complete and detailed statistical information is provided in Supplementary Table 1.

For behavioral pattern separation, two relevant LDR Test metrics—time to reach the first reversal and percent correct to the first reversal—were measured in both groups with Large and Small separations from Block 1 and Block 6 (Figures 4A–F). In Block 1, the SCR shRNA and TRIP8b shRNA mice took a similar amount of time to reach the first reversal (Figure 4C, Supplementary Table 1) with similar accuracy (Figure 4D, Supplementary Table 1) in both Large and Small separations. In Block 6, the SCR shRNA and TRIP8b shRNA mice also took a similar amount of time to the first reversal in Large separation. However, in Block 6 Small separation, there was a treatment effect on both speed and accuracy: The TRIP8b shRNA mice took 43% less time vs. the SCR shRNA mice to reach the first reversal (Figure 4E, Supplementary Table 1), and the TRIP8b shRNA mice were 45% more accurate vs. the SCR shRNA mice (Figure 4F, Supplementary Table 1). Of note, there was no treatment effect on the number of trials to the first reversal (data not shown); thus, TRIP8b shRNA mice were faster to get to the first reversal but did so in the same number of trials. These data suggest in the last LDR Test that LEC TRIP8b knockdown enhances behavioral pattern separation when stimuli locations are challenging to differentiate, and thus the cognitive load is high (Yassa et al., 2011; Bekinschtein et al., 2013; Kent et al., 2015a,b; Kassab and Alexandre, 2018).

### LEC TRIP8b shRNA mice have improved cognitive flexibility compared to SCR shRNA mice

In the LDR Test, cognitive flexibility over time can be inferred by the number of reversals made in Block 1 (Figure 4G) and Block 6 (Figure 4H), respectively. In Block 1, there was no treatment effect on the reversal number. There was, however, a main effect of separation. A *post hoc* analysis of Block 1 showed that the SCR shRNA mice made fewer reversals in Small separation vs. Large separation (Figure 4G, Supplementary Table 1) reflecting the difficulty of Small separation (high load) vs. Large separation (low load). For Block 6, there was a treatment effect in Block 6 Small separation: while only approaching significance (*p* = 0.08, but medium effect size), the TRIP8b shRNA mice accomplished 260% more reversals vs. the SCR shRNA mice (Figure 4H). This suggests that LEC TRIP8b knockdown improved cognitive flexibility under high load.

### Improved behavioral pattern separation is seen in mice where TRIP8b shRNA is expressed in LEC → DG Mol neurons but not in LEC → DG Mol + CA1 SLM neurons

Axon terminals from stellate cells in the LEC and MEC terminate in the outer Mol of DG and also in non-DG regions, such as the SLM of CA1, respectively (Witter, 2007; Kohara et al., 2014; Kitamura et al., 2015; Witter et al., 2017; Vandrey et al., 2020). All TRIP8b shRNA data presented up to this point reflect only “on target” mice where the expression of GFP+ was largely restricted to LEC → DG Mol neurons: GFP+ cell bodies in the LEC layer IIa (largely excluded from layer IIb) and GFP+ processes and terminals in DG Mol, but not in CA1 SLM (Supplementary Figures 1B, D). To assess if the TRIP8b shRNA-induced improvement in behavioral pattern separation and cognitive flexibility was restricted to only these “on target” LEC → DG Mol mice, we compared behavioral output among TRIP8b shRNA LEC → DG Mol, SCR shRNA mice, and TRIP8b shRNA LEC → DG Mol+CA1 mice (where GFP+ cell bodies were seen in LEC IIb and GFP+ processes and terminals were seen in SLM CA1; Supplementary Figures 1C, D). In Block 6 under conditions of Large separation, measures of behavioral pattern separation (time to the first reversal and percent correct to the first reversal; Supplementary Figures 1E, F) and cognitive flexibility (number of reversals achieved; Supplementary Figure 1G) were similar among all three groups of mice (SCR shRNA, TRIP8b shRNA LEC → DG Mol, and TRIP8b shRNA LEC → DG Mol+CA1 mice). However, in Block 6 under conditions of Small separation, TRIP8b shRNA LEC → DG Mol mice reached the first reversal faster (Supplementary Figure 1H) and had greater accuracy in the first reversal (Supplementary Figure 1I) compared to SCR shRNA mice, suggesting improved behavioral pattern separation. There was a visual difference in these measures of behavioral pattern separation between the SCR shRNA mice and TRIP8b shRNA LEC → DG Mol+CA1 mice, but the difference was rejected by one-way ANOVA. In contrast to the improved behavioral pattern separation in TRIP8b shRNA LEC → DG Mol mice vs. SCR shRNA mice, a parameter of cognitive flexibility (number of reversals) was not different among the three groups of mice (Supplementary Figure 1J).

### The improved behavioral pattern separation and cognitive flexibility seen in LEC TRIP8b shRNA mice are not a reflection of altered attention, motivation, or impulsivity

**Figure 5:**
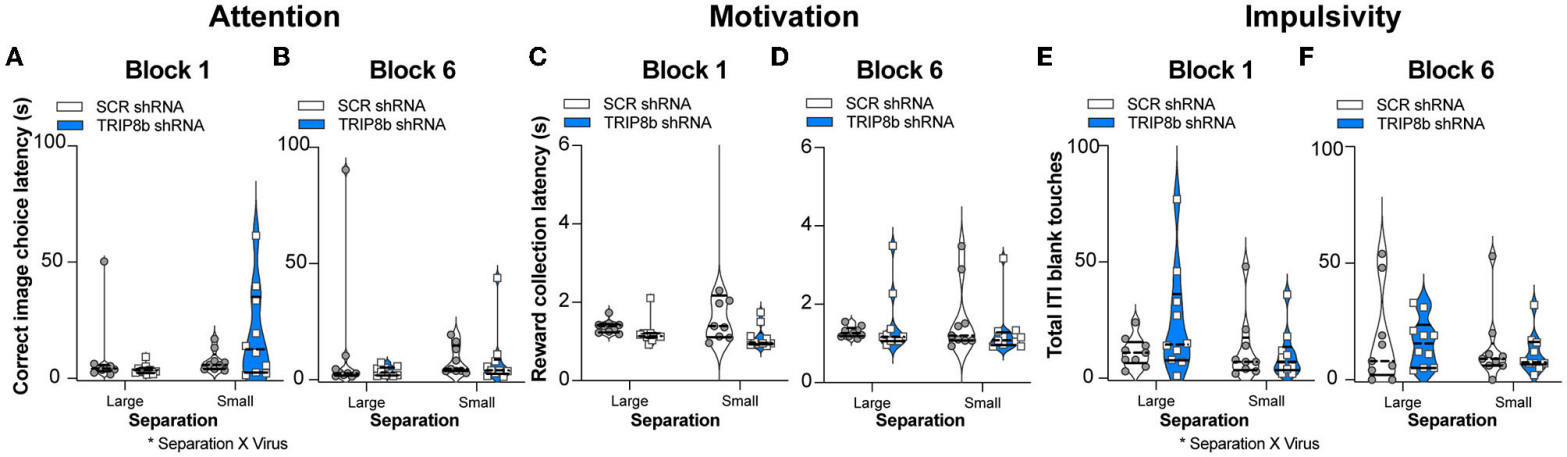
Knockdown of TRIP8b in the LEC did not change measures relevant to attention, motivation, or impulsivity during the LDR Test. **(A–F)** TRIP8b shRNA mice and SCR shRNA mice performed similarly on the following measures collected from Block 1 **(A, C, E)** or Block 6 **(B, D, F)** when faced with a stimuli separation that was either Large or Small in LDR Test: **(A, B)** Latency to choose the correct image; **(C, D)** Latency to collect the reward from the food hopper; and **(E, F)** Number of touches to a blank screen during the intertrial interval (ITI). Two-way RM ANOVA for all panels. **(A)** Main effects: Separation *F* (1, 17) = 2.977, *p* = 0.1026 and Treatment *F* (1, 17) = 0.4937, *p* = 0.4918, *post hoc*: *p* = 0.1204 in SCR vs. TRIP8b on Small separation; Interaction: Separation x Treatment *F* (1, 17) = 4.610, *p = 0.0465. **(B)** Main effects: Separation *F* (1, 17) = 0.0003390, *p* = 0.9855 and Treatment *F* (1, 17) = 0.8853, *p* = 0.3599; Interaction: Separation x Treatment *F* (1, 17) = 0.8399, *p* = 0.3722. **(C)** Main effects: Separation *F* (1, 17) = 1.144, *p* = 0.2997 and Treatment *F* (1, 17) = 2.248, *p* = 0.1521; Interaction: Separation x Treatment *F* (1, 17) = 1.539, *p* = 0.2316. **(D)** Main effects: Separation *F* (1, 17) = 0.09015, *p* = 0.7676 and Treatment *F* (1, 17) = 0.1240, *p* = 0.7291; Interaction: Separation x Treatment *F* (1, 17) = 1.383, *p* = 0.2559. **(E)** Main effects: Separation *F* (1, 17) = 3.077, *p* = 0.0974 and Treatment *F* (1, 17) = 0.7417, *p* = 0.4011, *post hoc: p* = *0.1537* in SCR vs. TRIP8b on Large separation; Interaction: Separation x Treatment *F* (1, 17) = 4.547, *p = 0.0478. **(F)** Main effects: Separation *F* (1, 17) = 1.877, *p* = 0.1885 and Treatment *F* (1, 17) = 0.08871, *p* = 0.7694; Interaction: Separation x Treatment *F* (1, 17) = 0.0446, *p* = 0.8353. LDR, location discrimination reversal; s, seconds. Complete statistical information is provided in Supplementary Table 1.

Circuit-based manipulations can influence the LDR Test performance by indirectly changing attention, motivation, or impulsivity. To test this possibility, data were extracted from the LDR Test sessions to assess latency to select a correct image, latency to collect the reward from the food hopper, and total number of blank touches made during the ITI which are measures of attention, motivation, and impulsivity, respectively. These LDR Test measures were collected from both Block 1 and Block 6 and under Large and Small separation conditions (Figure 5). While there was no treatment effect in any metric or condition (Figures 5A–F), there were two interactions (Separation x Treatment): correct image choice latency (Figure 5A) and ITI blank touches (Figure 5E). However, *post hoc* analysis did not reveal any difference. Thus, changes in attention, motivation, or impulsivity did not contribute to the improved behavioral pattern separation and cognitive flexibility seen in the TRIP8b shRNA vs. SCR shRNA mice.

### LEC TRIP8b shRNA and SCR shRNA mice have similar exploration and performance in a task of innate anxiety

**Figure 6:**
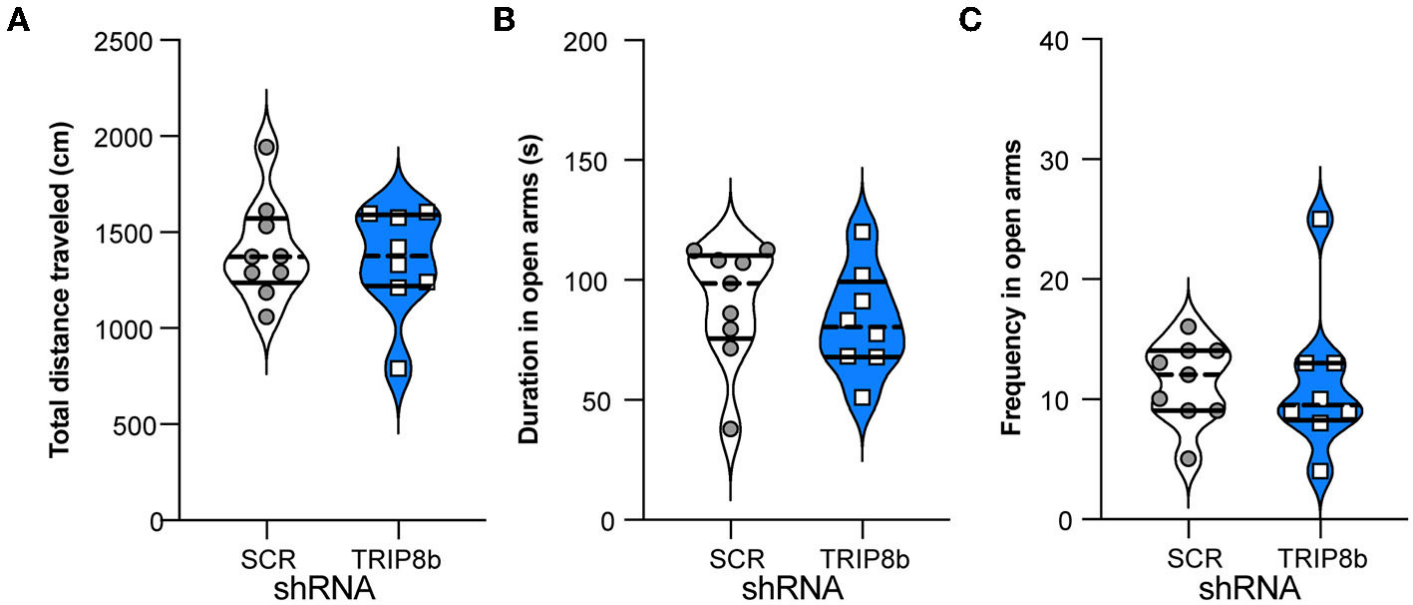
Knockdown of TRIP8b in the LEC does not induce anxiolytic-like behavior. **(A)** SCR shRNA mice and TRIP8b shRNA mice explore similarly in a novel environment, as based on a total movement traveled. **(B, C)** SCR shRNA mice and TRIP8b shRNA mice have similar performance in the elevated plus maze, based on time spent in open arms **(B)** and frequency to enter open arms **(C)**. Unpaired *t*-test for all panels: **(A)** *p* = 0.6529, **(B)** *p* = 0.5044, **(C)** *p* = 0.9863. Complete statistical information is provided in Supplementary Table 1.

Another indirect way that circuit-based manipulations can influence LDR Test performance is by changing measures relevant to exploration and innate anxiety. For example, perhaps the TRIP8b shRNA mice have a shorter time to the first reversal in the LDR Test because they are also less anxious. This possibility was tested by assessing mice in the EPM (Figure 1A). Consistent with prior work (Yun et al., 2018), the TRIP8b shRNA and SCR shRNA mice moved a similar total distance (Figure 6A), spent a similar amount of time in the open EPM arms (Figure 6B), and entered the open arms at a similar frequency (Figure 6C). These data suggest that TRIP8b shRNA-induced changes in innate exploration and anxiety did not contribute to TRIP8b-induced improvement in behavioral pattern separation and cognitive flexibility.

### LEC TRIP8b shRNA mice have more DG neurogenesis compared to SCR shRNA mice

**Figure 7:**
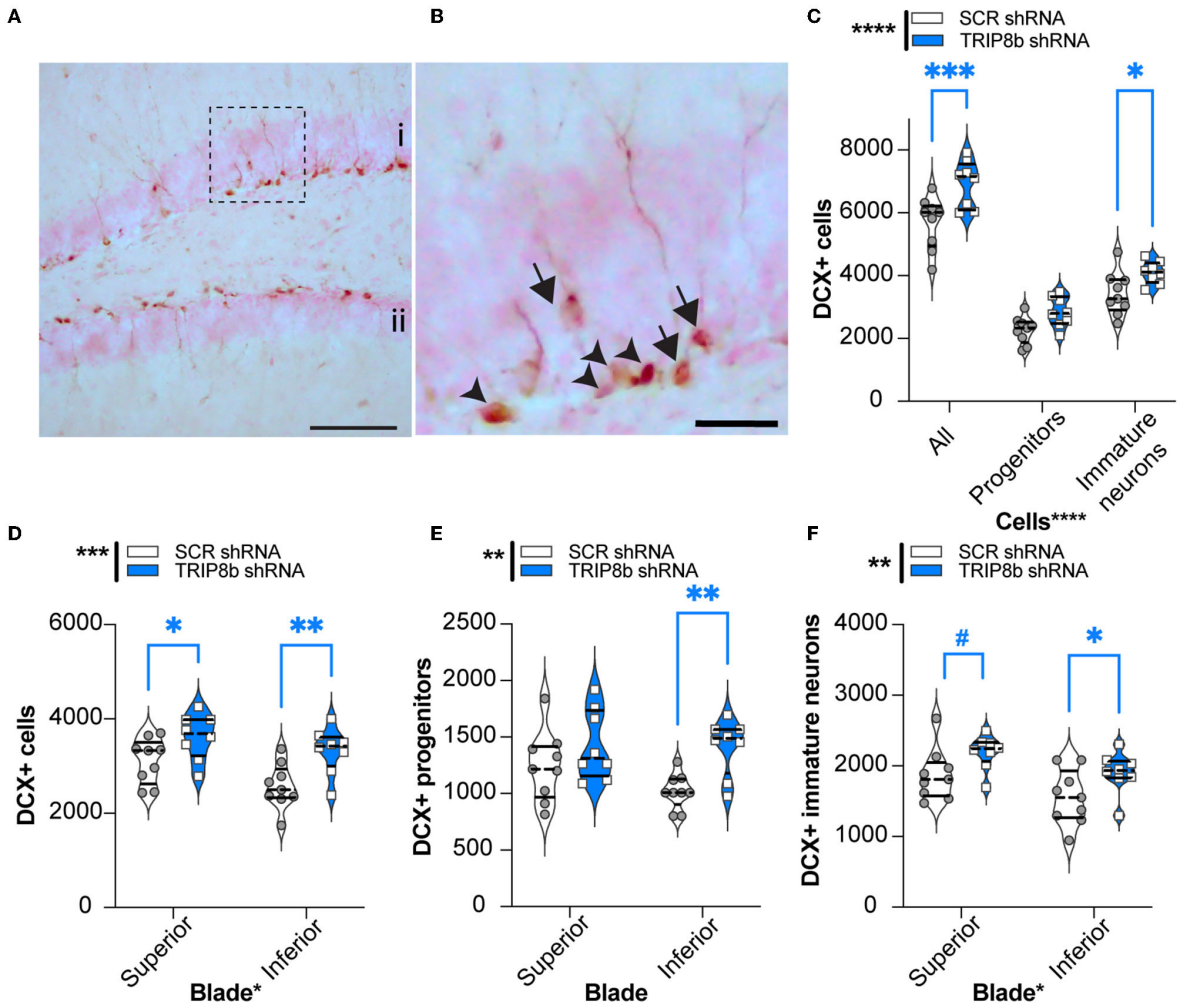
Knockdown of TRIP8b in the LEC increases DCX-immunoreactive (+) cells, an index of dentate gyrus adult neurogenesis. **(A, B)** Representative microscopic images of DCX cells from dorsal DG [i: superior blade, ii: inferior blade in **(A)**]. **(B)** High magnification images for cell types from the dotted square in A (Arrowheads: progenitor cells and arrows: immature cells). **(C)** TRIP8b KD in the LEC increases the number of total DCX+ cells and DCX+ immature neurons in the dorsal DG (−0.95 to −2.65 mm). **(B)** TRIP8b KD in the LEC increases DCX+ immature neurons in both the suprablade and infrablade of the DG. **(C)** TRIP8b KD in the LEC increases the number of DCX+ late progenitors in the infrablade. Two-way ANOVA for all panels: Main Effects: **(C)** Virus *F* (1, 45) = 23.58, ****p < 0.0001, *post-hoc*: *p = 0.0292 in SCR vs. TRIP8b in immature neurons and Cell type *F* (2, 45) = 165.4, ****p < 0.0001; interaction: Virus X Cell type *F* (2, 45) = 1.466, *p* = 0.2416. **(D)** Virus *F* (1, 30) = 14.50, ****p = 0.0006, *post-hoc*: *p = 0.0237 in SCR vs. TRIP8b in superior, **p = 0.0032 SCR vs. TRIP8b in inferior; and region *F* (1, 0) = 6.466, *p = 0.0164 interaction: Virus X Region *F* (1, 30) = 0.5293, *p* = 0.4726. **(E)** Virus *F* (1, 30) = 10.55, **p = 0.0029, post hoc: **p = 0.0041 in SCR vs. TRIP8b in the inferior region, and region *F* (1, 30) = 1.712, *p* = 0.2006; interaction: Virus X Region *F* (1, 30) = 1.330, *p* = 0.2579. **(F)** Virus *F* (1, 30) = 8.813, **p = 0.0058, *post-hoc*: #*p* = 0.0548 in SCR vs. TRIP8b in the superior region, *p = 0.0357 in SCR vs. TRIP8b in the inferior region, and region *F* (1, 30) = 7.121, *p* = 0.0122; interaction: Virus X Region *F* (1, 30) =0.0202, *p* = 0.8878. Scale bars: 100 um **(A)**, 25 um **(B)**. Complete statistical information is provided in Supplementary Table 1.

Manipulations that increase EC activity, including EC TRIP8b knockdown, increase indices of DG neurogenesis (Stone et al., 2011). As increased DG neurogenesis is linked to improved behavioral pattern separation and cognitive flexibility (Bekinschtein et al., 2011; Burghardt et al., 2012; McAvoy and Sahay, 2017), we hypothesized that LEC TRIP8b shRNA mice would have more DG neurogenesis compared to SCR shRNA mice. The brains from a random subset of mice that underwent behavioral assessment (Figure 1A) were assessed for the number of DCX+ cells in the dorsal DG, and those DCX+ cells were categorized via morphology as progenitor cells or immature neurons (Figures 7A, B). There were treatment effects for all neurogenesis measures (Figures 7C–F). Compared to the SCR shRNA mice, the TRIP8b shRNA mice had ~22% more total DCX+ cells and 20% more immature neurons, with no change in the number of progenitors (Figure 7C). We also considered the DCX+ cell counts in the superior vs. inferior blades of the DG as there are functional differences between these aspects of the DG GCL (Collins et al., 2009; Jinno, 2011; Raber et al., 2011; Alves et al., 2018; Raven et al., 2019). Compared to the SCR shRNA mice, the TRIP8b shRNA mice had 16% and 30% more DCX+ cells in the superior and inferior blades, respectively (Figure 7D). Based on morphology and compared to the SCR shRNA mice, the TRIP8b shRNA mice had ~38% more DCX+ progenitor cells only in the inferior blade (Figure 7E), and 17% and ~22% more DCX+ immature neurons in the superior and inferior blades, respectively (Figure 7F).

## Discussion

Improved understanding of the cell- and circuit-based function of LEC → DG projections will provide insight into many aspects of biomedical research, including cognitive decline in normal aging, disease progression in Alzheimer's disease, and neurostimulation to combat cognitive dysfunction or other neuropsychiatric symptoms (Albert, 1996; Stranahan and Mattson, 2010; Shin et al., 2014; Zhou et al., 2016; Bernstein and McNally, 2018; Leal and Yassa, 2018; Yu et al., 2019; Igarashi, 2022). Before this current study, few studies had examined how projections from the EC specifically to the hippocampal DG subregion—for example, LEC → DG projections—influence behavior. Here, we focused on LEC fan cells for three reasons: they are the only LEC glutamatergic neuron type that directly projects to the DG, they synapse on DG neurons critical for discrimination and reversal learning (e.g., granule and adult-generated neurons), and they are responsible for the formation of complex associations required for these cognitive abilities. We designed our study to complement prior studies. For example, one prior study showed that ablation of LEC fan cells projecting to the DG in male mice decreased discrimination of novel object-place-context configurations, but did not impact novel object or object-context recognition (Vandrey et al., 2020). A less cell-type specific lesion study showed that intact LEC and PRH are required for optimal performance in the LDR behavioral pattern separation paradigm (Vivar et al., 2012). Also, a transection study showed that an intact perforant path (which includes, but is not specific to, LEC → DG projections) is required for object-based behavioral pattern separation (Burke et al., 2018). As prior work on the function of LEC → DG projections employed lesion strategies, here we asked if the activity of LEC → DG projections influences behavioral pattern separation in male mice. We also probed if LEC → DG projection activity regulates cognitive flexibility. We hypothesized that increased activity of LEC → DG neurons would improve the relatively complex abilities of behavioral pattern separation and reversal learning as EC-DG stimulation does with simpler, one-trial hippocampal-dependent associative learning (Yun et al., 2018). We report that relative to control mice, male mice that received LEC TRIP8b shRNA showed improved indices of both behavioral pattern separation and cognitive flexibility. The *post-mortem* analysis showed that they also had more DG neurogenesis, supporting that our manipulation increased DG activity, as shown in prior work (Yun et al., 2018). Our findings clarify the circuitry engaged in these higher cognitive abilities and are novel in that they implicate the activity of the “upstream” LEC in behavioral pattern separation and cognitive flexibility.

There are three particularly notable aspects of the data presented here. First, long-term stimulation of LECIIa fan cells improves measures of location discrimination when the load on pattern separation is high (lit squares are close together) but not when the load is low (lit squares are far apart). Testing discrimination under different loads is a defining feature of behavioral pattern separation paradigms (Rolls and Kesner, 2006). This finding was expected; the DG is critical for behavioral pattern separation (Leutgeb et al., 2007; Bakker et al., 2008; Clelland et al., 2009) and increased activity of the EC → DG pathway improves DG-dependent behavioral tasks (Stone et al., 2011; Xia et al., 2017; Yun et al., 2018). However, this finding is important. In showing that increased activity of the LEC → DG circuit improves behavioral pattern separation, this finding suggests that DG dysfunction may be reversed by targeting the activity of afferent regions rather than targeting the DG itself (Suthana et al., 2012; Jacobs et al., 2016).

Second, long-term stimulation of LECIIa fan cells also improves measures of reversal learning. Cognitive flexibility is typically considered to be regulated by the activity and integrity of the prefrontal cortex (PFC) (Kehagia et al., 2010; Izquierdo et al., 2017; Girotti et al., 2018). However, many studies point to a role of the DG in cognitive flexibility (Burghardt et al., 2012; Swan et al., 2014; Lucassen and Oomen, 2016; Anacker and Hen, 2017; Berdugo-Vega et al., 2021; Gomes-Leal, 2021). For example, the ablation of DG adult-generated neurons decreases cognitive flexibility in the LDR task (Swan et al., 2014). Our data now show a role for the activity of the upstream EC in cognitive flexibility. The specific mechanism and circuitry underlying this effect are not tested in the present study. One consideration for future research is that induced stimulation of LECIIa fan cells (which project to the DG) may indirectly alter the activity of LEC cells that project to other brain regions (such as the LECII pyramidal cells or neurons in deep LEC layers projecting to the PFC) or even of MEC cells (Insausti et al., 1997; Delatour and Witter, 2002; Tanji and Hoshi, 2008; Canto and Witter, 2012; Ohara et al., 2020; Yu et al., 2021).

A final notable aspect of our data is the timing of the effect. We show long-term stimulation of LECIIa fan cells improves indices of behavioral pattern separation (performance before the first reversal) and cognitive flexibility (performance after first reversal) in the last block, but not in the first block, of LDR Test, and not during the general touchscreen training or LDR training. As we did not examine the brains of the mice during the first and last blocks of the LDR Test, we can only speculate why the TRIP8b-induced improvement in behavioral pattern separation and cognitive flexibility only emerges with time. One possibility is that the increased LEC → DG circuit activity increases DG neurogenesis which over time contributes to improved behavioral pattern separation and cognitive flexibility. Indeed, the start of the LDR Test was designed to coincide with a timepoint post-AAV infusion when both induced gene expression is maximal (4 weeks) and DG neurogenesis is increased after EC infusion of TRIP8b shRNA (Yun et al., 2018). The speculative causal involvement of DG neurogenesis is supported by prior work where induced ablation of postnatal DG neurogenesis (which is essentially a “long-term” manipulation) impaired cognitive flexibility in the last block, but not the first block, of LDR (Swan et al., 2014). Our current data on DCX+ neuron numbers provide correlative insight into this speculation. DCX+ neuron number is influenced by LEC → DG circuit activity and also by LDR performance, and it is not possible to dissociate these two influences. Direct testing of this hypothesis is possible in the future, however. For example, both long-term stimulation of the LEC → DG circuit (present study) and ablation of DG cells/EC circuit lesions (prior work) likely result in compensatory circuit changes (Vivar et al., 2012; Swan et al., 2014; Vandrey et al., 2020). Long-term—but not acute—EC stimulation improves DG-dependent behavior (Yun et al., 2018), yet it remains to be tested if a single stimulation followed by time improves DG-dependent behavior, as has been shown with acute manipulation of neurogenesis (Airan et al., 2007; Xia et al., 2017). An alternative way to test if LEC → DG circuit stimulation increases DG neurogenesis and improves pattern separation and cognitive flexibility is to employ a behavioral pattern separation task that can be performed over a few days (spontaneous location recognition [SLR] or object lure discrimination and mnemonic discrimination testing) rather than a month (LDR, used in the present study). Using a time-condensed behavioral pattern separation task such as SLR would also enable future dissociation of the stages of learning (encoding, consolidation, and retrieval) and factors that regulate each stage (Bekinschtein et al., 2013; Johnson et al., 2018; Morales et al., 2021; Reichelt et al., 2021).

This study adds to the already known role of human and rodent EC → hippocampal projections in learning and memory (Yassa et al., 2010; Stone et al., 2011; Wilson et al., 2013; Hansen et al., 2018; Amani et al., 2021), antidepressant-like behavior (Yun et al., 2018), and reward-seeking (Ge et al., 2017). This study also raises interesting questions. For example, although our study shows that increased activity of LEC → DG projections does not change the motivation for an operant reward, other studies show a role for midbrain dopamine (a neurotransmitter often linked to salience and reward) in EC associative memory encoding (Lee et al., 2021). Future research is warranted to learn if the activity of LEC → DG projections also regulates behavioral pattern separation and cognitive flexibility in the context of animal states and traits, such as animal models for addiction, which are marked by altered midbrain dopaminergic neuron activity (Schultz, 1997; Ungless et al., 2010; Morikawa and Paladini, 2011; Marinelli and McCutcheon, 2014; Keiflin and Janak, 2015; Wise and Jordan, 2021), and whether different EC → DG or EC → hippocampal circuits are engaged under different conditions (Kassab and Alexandre, 2018). Furthermore, while the LEC → DG projections stimulated in the present study are glutamatergic, GABAergic EC → hippocampus projections also exist (Melzer et al., 2012; Caputi et al., 2013; Basu et al., 2016). These long-range inhibitory EC → hippocampus neurons are positioned to disinhibit hippocampal activity as they synapse on hippocampal GABAergic neurons. Thus, it would be interesting to see how the modulated activity of long-range inhibitory EC → hippocampal projections influences behavioral pattern separation and cognitive flexibility.

In sum, the present data show that altered activity of male mouse LEC → DG projections regulates both behavioral pattern separation and cognitive flexibility. This study provides insight into the cellular and circuit mechanisms underlying these cognitive abilities and opens avenues for developing circuit-based treatments for impaired hippocampal cognition. As such, these data advance fundamental and translational neuroscience knowledge relevant to two cognitive functions critical for adaptation and survival.

## Data availability statement

The original contributions presented in the study are included in the article/Supplementary material. Further inquiries can be directed to the corresponding authors.

## Ethics statement

The animal study was reviewed and approved by the Institutional Animal Care and Use Committee at the Children's Hospital of Philadelphia (CHOP) and performed in compliance with the National Institutes of Health Guide for the Care and Use of Laboratory Animals.

## Author contributions

Conceptualization: SY, AE, and RR. Methodology: IS, SY, FT, MS, RR, and AE. Software: RS and GB. Validation: SY, HH, GB, and AE. Formal analysis: IS, FT, SY, HH, and GB. Investigation: IS, SY, FT, MS, CSD, RR, and HH. Resources, writing—original draft, supervision, projection administration, and funding acquisition: SY and AE. Data curation: IS, SY, HH, and GB. Writing—review and editing: SY, AE, and HH. Visualization: SY and HH. All authors contributed to the article and approved the submitted version.

## Supplementary material

Supplementary Figure 1 Relationship between hippocampal terminal regions expressing viral-mediated green fluorescent protein (GFP) and performance in LDR test. **(A–D)** 14 weeks after bilateral LEC stereotaxic infusion of AAV-TRIP8bshRNA-EGFP or AAV-SCRshRNA-EGFP and <4 days after the final behavioral test, GFP-immunoreactive (GFP+) cell bodies were detected in LEC Layer IIa **(A)**, a layer enriched with stellate cells that project to the dentate gyrus molecular layer, DG Mol, and IIb **(C)**, a layer enriched with pyramidal cells that project to the CA1 stratum lacunosum molecular [SLM]). In mice in which the LECIIa was targeted, GFP+ terminals were detected in the outer DG Mol **(B)**; such mice were termed LEC = >DG Mol mice. If the virus also spread to LECIIb, then GFP+ terminals were evident in both the DG Mol as well as the CA1 SLM **(D)**; these mice were termed LEC → DG Mol+CA1 SLM mice. Data in the main text figures are all from LEC → DG Mol mice (mice with GFP+ terminal expression in the DG Mol but no expression in the CA1 SLM). In **(E–J)**, behavioral data are presented showing SCR shRNA mice and TRIP8b shRNA mice from both LEC → DG Mol mice and LEC → DG Mol+CA1 SLM mice. In Large separation, SCR shRNA and TRIP8b shRNA mice (both LEC → DG Mol and LEC → DG Mol+CA1 SLM) had similar LDR Test measures, including **(E)** time to the first reversal, **(F)** % correct to the first reversal, and **(G)** reversal #. However, in Small separation, TRIP8b shRNA LEC = >DG Mol mice had better pattern separation vs. SCR shRNA based on **(H)** time to first reversal and **(I)** % correct to the first reversal but performed similarly in reversal learning **(J)**. Dotted lines delineate **(A, C)** IIa and IIb and **(B, D)** DG granule cell layer (GCL). Scale bar **(A)** = 200 um applies **(A-D)**. One-way ANOVA was used for all. **(E)** Main Effect: Treatment F (2.21) = 0.3079, *p* = 0.7383. **(F)** Main Effect: Treatment F (2.21) = 0.4144, *p* = 0.6660. **(G)** Main Effect: Treatment F (2, 21) = 0.8267, *p* = 0.4512. **(H)** Main Effect: Treatment F (2.21) = 6.406, ^**^*p* = 0.0067, *post-hoc*: ^**^*p* = 0.0068 in SCR vs TRIP8b mice (LEC → DG Mol mice). **(I)** Main Effect: Treatment F (2.21) = 3.496, ^*^*p* = 0.0489, *post-hoc*: ^*^*p* = 0.0472 in SCR vs. TRIP8b mice (LEC → DG Mol mice). **(J)** Main Effect: Treatment F (2.21) = 2.882, *p* = 0.0783. Complete statistical information is provided in Supplementary Table 1.

Supplementary Table 1 Statistical analyses for each figure panel and all results.
