# Supplementary figures and images for: Behavioral pattern separation and cognitive flexibility are enhanced in a mouse model of increased lateral entorhinal cortex-dentate gyrus circuit activity
Source: Front Behav Neurosci. 2023 Jun 1;17:1151877. doi: 10.3389/fnbeh.2023.1151877 (PMC10267474; doi:10.3389/fnbeh.2023.1151877)

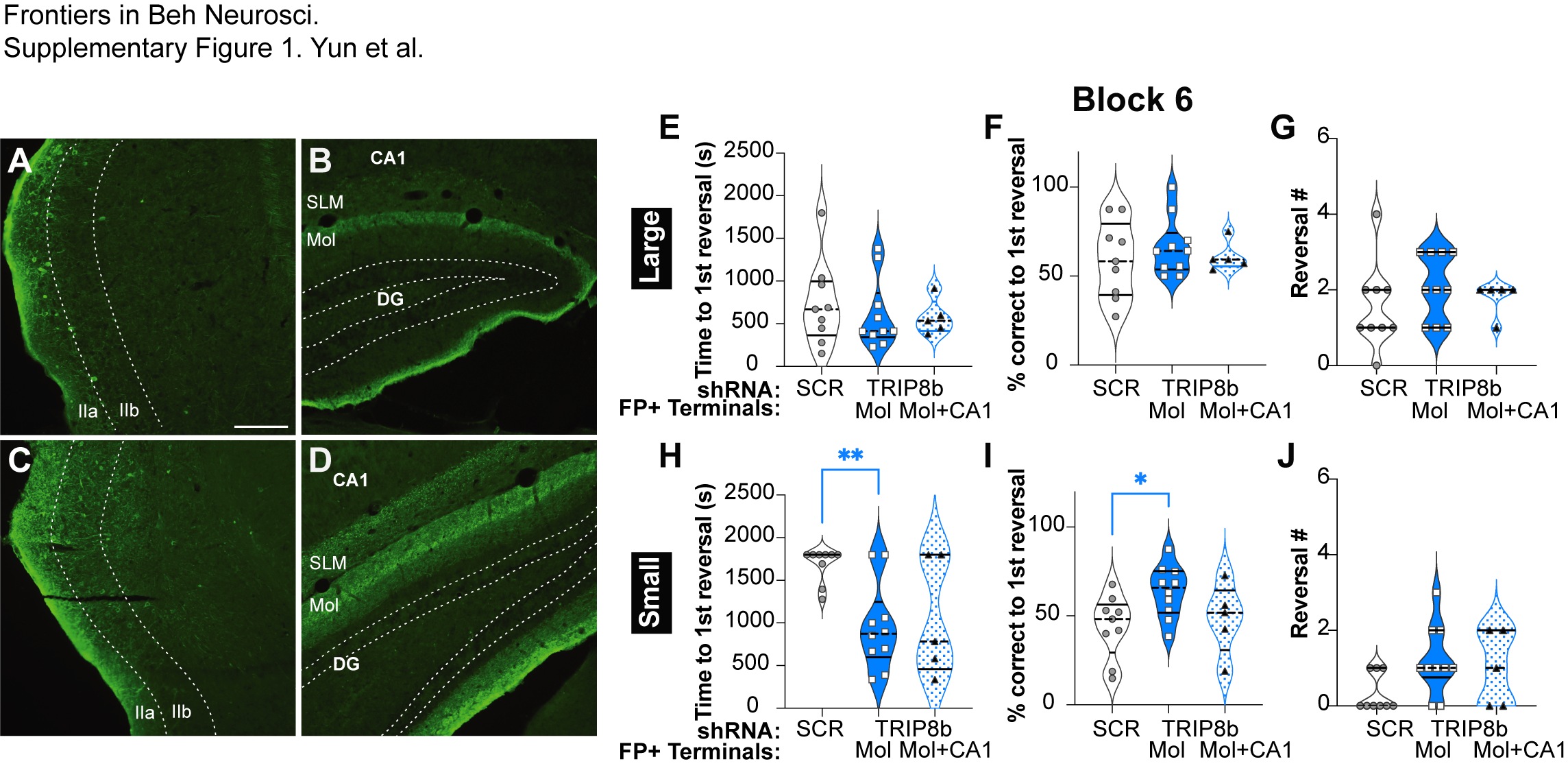

Supplement: Supplementary Figure 1 — Relationship between hippocampal terminal regions expressing viral-mediated green fluorescent protein (GFP) and performance in LDR test. (A–D) 14 weeks after bilateral LEC stereotaxic infusion of AAV-TRIP8bshRNA-EGFP or AAV-SCRshRNA-EGFP and <4 days after the final behavioral test, GFP-immunoreactive (GFP+) cell bodies were detected in LEC Layer IIa (A), a layer enriched with stellate cells that project to the dentate gyrus molecular layer, DG Mol, and IIb (C), a layer enriched with pyramidal cells that project to the CA1 stratum lacunosum molecular [SLM]). In mice in which the LECIIa was targeted, GFP+ terminals were detected in the outer DG Mol (B); such mice were termed LEC = >DG Mol mice. If the virus also spread to LECIIb, then GFP+ terminals were evident in both the DG Mol as well as the CA1 SLM (D); these mice were termed LEC → DG Mol+CA1 SLM mice. Data in the main text figures are all from LEC → DG Mol mice (mice with GFP+ terminal expression in the DG Mol but no expression in the CA1 SLM). In (E–J), behavioral data are presented showing SCR shRNA mice and TRIP8b shRNA mice from both LEC → DG Mol mice and LEC → DG Mol+CA1 SLM mice. In Large separation, SCR shRNA and TRIP8b shRNA mice (both LEC → DG Mol and LEC → DG Mol+CA1 SLM) had similar LDR Test measures, including (E) time to the first reversal, (F) % correct to the first reversal, and (G) reversal #. However, in Small separation, TRIP8b shRNA LEC = >DG Mol mice had better pattern separation vs. SCR shRNA based on (H) time to first reversal and (I) % correct to the first reversal but performed similarly in reversal learning (J). Dotted lines delineate (A, C) IIa and IIb and (B, D) DG granule cell layer (GCL). Scale bar (A) = 200 um applies (A-D). One-way ANOVA was used for all. (E) Main Effect: Treatment F (2.21) = 0.3079, p = 0.7383. (F) Main Effect: Treatment F (2.21) = 0.4144, p = 0.6660. (G) Main Effect: Treatment F (2, 21) = 0.8267, p = 0.4512. (H) Main Effect: Treatment F (2.21) = 6.406, **p = 0.00 [file Image_1.tif]
