# Supplementary material for: Behavioral pattern separation and cognitive flexibility are enhanced in a mouse model of increased lateral entorhinal cortex-dentate gyrus circuit activity
Source: Front Behav Neurosci. 2023 Jun 1;17:1151877. doi: 10.3389/fnbeh.2023.1151877 (PMC10267474; doi:10.3389/fnbeh.2023.1151877)
Supplement: Supplementary Table 1 — Statistical analyses for each figure panel and all results. [file Table_1.pdf]

Supp Table: Yun et al., Frontier Beh neuroscience

Supplementary Table. Statistical analyses for each figure panel and all results.

Bold text and ***, p<0.05. Italicized text, 0.05<p<0.1. *Magnitudes of partial omega-squared (for RM two-way ANOVA): 0.01 small; 0.06 medium; 0.14 large. N/A not applicable.

Dependent Measure	Figure Panel	Group: Subject Number	Mean	Statistical Test	Variables, Main Effect, Interaction *P<0.5 **P<0.01 ***P<0.001 ****P<0.0001	F Value	P value	Post hoc Test (Bonferroni for 2-way ANOVA, Tukey for 1-way ANOVA)	Effect size (when RM two-way ANOVA p<0.05, partial omega-squared is calculated where ≤0.05 small, ≥0.06 medium, ≥0.14 large)	CI (95%)
Weights	1D		Post-surgery (wks): 0, 1, 2, 3, 4, 5, 6, 7, 8, 9, 10, 11, 12, 13, 14	RM 2-way ANOVA	Time****					
		SCR: 9	SCR: 25.2, 25.77, 24.71, 24.98, 25.46, 25.9, 26.28, 26.36, 26.76, 26.96, 27.21, 27.14, 27.51, 28.04, 32.04			F (14, 238) = 130.6	**P<0.0001**	N/A		
		TRIP8b: 10	TRIP: 24.43, 25.98, 24.27, 24.54, 24.62, 24.95, 25.45, 25.5, 26.21, 26.21, 26.78, 26.93, 27.1, 27.65, 31.41		Virus	F (1, 17) = 0.6704	P=0.4242			
					Interaction	F (14, 238) = 0.9406	P=0.5158			
General Touchscreen Training w/six windows	2A	SCR: 9	General TS training	RM 2-way ANOVA	Training Stage****					
			Training Stage: HAB, IT, MT, MI, PI			F (4, 68) = 75.03	**P<0.0001**	N/A	0.92	.77, .96
		TRIP8b: 10	SCR: 1, 1, 5.222, 1.111, 12.22		Virus	F (1, 17) = 0.6292	P=0.4386			
			TRIP: 1, 1, 4.4, 1.1, 10.9		Interaction	F (4, 68) = 0.3333	P=0.8547			
Punish Incorrect: Session Length	2B	SCR: 9	Session length (s)	RM 2-way ANOVA	Training Stage					
			Session: Day 1, Last			F (1, 17) = 0.08579	P=0.7731	N/A		
		TRIP8b: 10	SCR: 1292, 1256		Virus	F (1, 17) = 0.2492	P=0.6240			
			TRIP: 1349, 1312		Interaction	F (1, 17) = 2.128e-	P=0.9964			
Punish Incorrect: Trial Number	2C	SCR: 9	Trial #	RM 2-way ANOVA	Training Stage					
			Session: Day 1, Last			F (1, 17) = 2.858	P=0.1092	N/A		
		TRIP8b: 10	SCR: 29.89, 30		Virus	F (1, 17) = 2.335	P=0.1449			
			TRIP: 27.8, 30		Interaction	F (1, 17) = 2.335	P=0.1449			
Punish Incorrect: Percent Correct	2D	SCR: 9	% correct	RM 2-way ANOVA	Training Stage****					
			Session: Day 1, Last			F (1, 17) = 148.2	**P<0.0001**	Day 1 vs Last on both: ****p<0.0001	0.85	.64, .94
		TRIP8b: 10	SCR: 53.19, 90		Virus	F (1, 17) = 1.599	P=0.2231			
			TRIP: 61.82, 89		Interaction	F (1, 17) = 3.358	P=0.0844			
LDR Train: Percent reaching criteria	3B		LD train criteria completion curve	Log-rank (Mantel-Cox) test	N/A	N/A	p=0.5328	N/A		
			SCR: Median: 5.5							
			TRIP: Median: 7							
LDR Train: Days to completion	3C		Days to completion	Unpaired T-test	N/A	N/A	p=0.1088	N/A		
		SCR: 9	SCR: 5.111 ± 0.824							
		TRIP8b: 10	TRIP: 7.4 ± 1.046							
LDR Train: Percent correct	3D	SCR: 9	% correct	RM 2-way ANOVA						
			Session: Day 1, Last day		Day**	F (1, 17) = 10.08	**p=0.0055**	Day 1 vs Last day in TRIP8b: **p=0.0075	0.19	0.00, 0.53
		TRIP8b: 10	SCR: 39.29, 45.47		Virus	F (1, 17) = 0.00067	p=0.9796			
			TRIP: 34.87, 50.42		Interaction	F (1, 17) = 2.084	p=0.1671			
LDR Train: Time to 1st reversal	3E	SCR: 9	Time to 1st reversal	RM 2-way ANOVA						
			Session: Day 1, Last day		Day	F (1, 17) = 1.386	p=0.2552	N/A		
		TRIP8b: 10	SCR: 1316, 760		Virus	F (1, 17) = 1.500	p=0.2373			
			TRIP: 857.6, 932.3		Interaction	F (1, 17) = 2.382	p=0.1411			
LDR Train: Percent correct to 1st reversal	3F	SCR: 9	% correct to 1st reversal	RM 2-way ANOVA						
			Session: Day 1, Last day		Day	F (1, 17) = 1.571	p=0.2271	N/A		
		TRIP8b: 10	SCR: 47.92, 64.97		Virus	F (1, 17) = 2.700	p=0.1187			
			TRIP: 63.57, 68.08		Interaction	F (1, 17) = 0.5309	p=0.4762			
LDR Test: Block 1, time to 1st reversal	4C	SCR: 9	Block 1 time to 1st reversal (sec)	RM 2-way ANOVA						
			Session: Large, Small		Separation	F (1, 17) = 1.084	p=0.3124	N/A		
		TRIP8b: 10	SCR: 1032, 1460		Virus	F (1, 17) = 2.423	p=0.1380			
			TRIP: 1296, 1380		Interaction	F (1, 17) = 0.1799	p=0.6768			
LDR Test: Block 1, percent correct to 1st reversal	4D	SCR: 9	Block 1 % correct to 1st reversal	RM 2-way ANOVA	Separation					
			Session: Large, Small			F (1, 17) = 1.679	p=0.2123	N/A		
		TRIP8b: 10	SCR: 61.69, 47.32		Virus	F (1, 17) = 0.3233	p=0.5771			
			TRIP: 52.42, 50.03		Interaction	F (1, 17) = 0.8583	p=0.3672			
LDR Test: Block 6, time to 1st reversal	4E	SCR: 9	Block 6 time to 1st reversal (sec)	RM 2-way ANOVA						
			Session: Large, Small		Separation***	F (1, 17) = 16.36	**p=0.0008**	SCR vs TRIP8b in Small Separation: **p=0.0012	0.37	0.04, 0.68
		TRIP8b: 10	SCR: 730.6, 1685		Virus**	F (1, 17) = 15.53	**p=0.0011**		0.19	0.00, 0.53
			TRIP: 604.8, 950.5		Interaction	F (1, 17) = 3.589	p=0.0753			
LDR Test: Block 6, percent correct to 1st reversal	4F	SCR: 9	Block 6 % correct to 1st reversal	RM 2-way ANOVA	Separation					
			Session: Large, Small			F (1, 17) = 1.760	p=0.2022	SCR vs TRIP8b in Small Separation: *p=0.0397		
		TRIP8b: 10	SCR: 59.29, 44.03		Virus**	F (1, 17) = 8.939	**p=0.0082**		0.10	0.00, 0.44
			TRIP: 66.31, 63.76		Interaction	F (1, 17) = 0.8945	p=0.3575			
LDR Test: Block 1, reversal #	4G	SCR: 9	Block 1 reversal #	RM 2-way ANOVA	Separation					
			Session: Large, Small		**	F (1, 17) = 10.07	**p=0.0056**	Large Separation vs Small Separation in SCR: *p=0.0156	0.12	0.00, 0.46
		TRIP8b: 10	SCR: 1.444, 0.5556		Virus	F (1, 17) = 1.448	p=0.2454			
			TRIP: 0.8, 0.4		Interaction	F (1, 17) = 1.448	p=0.2453			
LDR Test: Block 6, reversal #	4H	SCR: 9	Block 6 reversal #	RM 2-way ANOVA	Separation					
			Session: Large, Small		**	F (1, 17) = 14.83	**p=0.0013**	SCR vs TRIP8b in Small separation: #p=0.0814	0.26	0.00, 0.59
		TRIP8b: 10	SCR: 1.556, 0.3333		Virus*	F (1, 17) = 5.531	**p=0.0310**		0.12	0.00, 0.46
			TRIP: 2.1, 1.2		Interaction	F (1, 17) = 0.3419	p=0.5664			
LDR Test: Block 1, correct image choice latency	5A	SCR: 9	Block 1 correct image choice latency (sec)	RM 2-way ANOVA						
			Session: Large, Small		Separation	F (1, 17) = 2.977	p=0.1026	SCR vs TRIP8b in Small separation: p=0.1204		
		TRIP8b: 10	SCR: 9.151, 7.497		Virus	F (1, 17) = 0.4937	p=0.4918			
			TRIP: 3.87, 19.06		Interaction*	F (1, 17) = 4.610	**p=0.0465**		0.08	0.00, 0.41
LDR Test: Block 6, correct image choice latency	5B	SCR: 9	Block 6 correct image choice latency (sec)	RM 2-way ANOVA						
			Session: Large, Small		Separation	F (1, 17) = 0.00033	p=0.9855	N/A		
		TRIP8b: 10	SCR: 13.24, 8.5		Virus	F (1, 17) = 0.8853	p=0.3599			
			TRIP: 3.68, 8.619		Interaction	F (1, 17) = 0.8399	p=0.3722			
LDR Test: Block 1, reward collection latency	5C	SCR: 9	Block 1 reward collection latency (sec)	RM 2-way ANOVA						
			Session: Large, Small		Separation	F (1, 17) = 1.144	p=0.2997	N/A		
		TRIP8b: 10	SCR: 1.376, 2.89		Virus	F (1, 17) = 2.248	p=0.1521			
			TRIP: 1.217, 1.105		Interaction	F (1, 17) = 1.539	p=0.2316			
LDR Test: Block 6, reward collection latency	5D	SCR: 9	Block 6 reward collection latency (sec)	RM 2-way ANOVA						
			Session: Large, Small		Separation	F (1, 17) = 0.09015	p=0.7676	N/A		
		TRIP8b: 10	SCR: 1.29, 1.632		Virus	F (1, 17) = 0.1240	p=0.7291			
			TRIP: 1.484, 1.281		Interaction	F (1, 17) = 1.383	P=0.2559			
LDR Test: Block 1, total ITI blank touches	5E	SCR: 9	Block 1 total ITI blank touches	RM 2-way ANOVA						
			Session: Large, Small		Separation	F (1, 17) = 3.077	p=0.0974	SCR vs TRIP8b in Large separation: p=0.1537		
		TRIP8b: 10	SCR: 11.33, 12.67		Virus	F (1, 17) = 0.7417	p=0.4011		0.05	0.00, 0.36
			TRIP: 24, 10.3		Interaction*	F (1, 17) = 4.547	**p=0.0478**			
LDR Test: Block 6, total ITI blank touches	5F	SCR: 9	Block 6 total ITI blank touches	RM 2-way ANOVA						
			Session: Large, Small		Separation	F (1, 17) = 1.877	p=0.1885	N/A		
		TRIP8b: 10	SCR: 17.11, 13.67		Virus	F (1, 17) = 0.08871	p=0.7694			
			TRIP: 16, 11.3		Interaction	F (1, 17) = 0.04460	p=0.8353			
EPM: Total distance traveled	6A		Total distance traveled	Unpaired t-test						
		SCR:9	1406		N/A	N/A	p=0.6529	N/A		
		TRIP8b: 8	1347							
EPM: Duration in open arms	6B		Duration in open arms	Unpaired t-test						
		SCR:9	90.46		N/A	N/A	p=0.5044	N/A		
		TRIP8b: 8	82.7							
EPM: Frequency in open arms	6C		Frequency in open arms	Unpaired t-test						
		SCR:9	11.33		N/A	N/A	p=0.9863	N/A		
		TRIP8b: 8	11.38							
Total DCX+ cells	7C		All cells, Progenitors, Immature Cells	2-way Anova	Cell type****	F (2, 45) = 165.4	**P<0.0001**			
		SCR:9	5685, 2260, 3426		Virus****	F (1, 45) = 23.58	**P<0.0001**	SCR vs TRIP8b in Immature Cells: p=0.0292	0.57	0.35, 0.73
		TRIP8b: 8	6936, 2846, 4098		Intreraction	F (2, 45) = 1.466	P=0.2416			
DCX+ cells (superior vs inferior)	7D		Superior, Inferior	2-way Anova	Regions*	F (1, 30) = 6.466	**P=0.0164**	SCR vs TRIP8B in superior region: p=0.0374		
		SCR:9	3113, 2572		Virus***	F (1, 30) = 14.50	**P=0.0006**	SCR vs TRIP8B in inferior region: p=0.0032	0.24	0.03, 0.51
		TRIP8b: 8	3622, 3322		Intreraction	F (1, 30) = 0.5293	P=0.4726			
DCX+ progenitor cells (superior vs inferior)	7E		Superior, Inferior	2-way Anova	Regions	F (1, 30) = 1.712	P=0.2006			
		SCR:9	1241, 1019		Virus**	F (1, 30) = 10.55	**P=0.0029**	SCR vs TRIP8b in inferior region: p=0.0041	0.36	0.10, 0.61
		TRIP8b: 8	1430, 1416		Intreraction	F (1, 30) = 1.330	P=0.2579			
DCX+ immature cells (superior vs inferior)	7F		Superior, Inferior	2-way Anova	Regions*	F (1, 30) = 7.121	**P=0.0122**	SCR vs TRIP8b in superior region: p=0.0548	0.31	0.07, 0.57
		SCR:9	1872, 1554		Virus**	F (1, 30) = 8.813	**P=0.0058**	SCR vs TRIP8b in inferior region: p=0.0357		
		TRIP8b: 8	2192, 1906		Intreraction	F (1, 30) = 0.02025	P=0.8878			
LDR Test: Block 6, Large separation, time to 1st reversal	Supp Fig 1E		Block 6 Large separation Time to 1st reversal (sec)	1-way ANOVA	Treatment	F (2, 21) = 0.3079	P=0.7383	N/A		
		SCR: 9	SCR: 730.6							
		TRIP8b (Mol): 10	TRIP 8b (Mol): 604.8							
		TRIP8b (Mol + CA1): 10	TRIP 8b (Mol + CA1): 575.8							
LDR Test: Block 6, Large separation, percent correct to 1st reversal	Supp Fig 1F		Block 6 Large separation % correct to 1st reversal	1-way ANOVA	Treatment	F (2, 21) = 0.4144	P=0.6660	N/A		
		SCR: 9	SCR: 59.29							
		TRIP8b (Mol): 10	TRIP 8b (Mol): 66.31							
		TRIP8b (Mol + CA1): 10	TRIP 8b (Mol + CA1): 60.91							
LDR Test: Block 6, Large separation, reversal number	Supp Fig 1G		Block 6 Large separation reversal #	1-way ANOVA	Treatment	F (2, 21) = 0.8267	P=0.4512	N/A		
		SCR: 9	SCR: 1.556							
		TRIP8b (Mol): 10	TRIP 8b (Mol): 2.1							
		TRIP8b (Mol + CA1): 10	TRIP 8b (Mol + CA1): 1.8							
LDR Test: Block 6, Small separation, time to 1st reversal	Supp Fig 1H		Block 6 Small separation Time to 1st reversal (sec)	1-way ANOVA	Treatment**	F (2, 21) = 6.406	**P=0.0067**	SCR vs TRIP8b (Mol): **p=0.0068	0.31	0.01, 0.58
		SCR: 9	SCR: 1685							
		TRIP8b (Mol): 10	TRIP 8b (Mol): 950.5							
		TRIP8b (Mol + CA1): 10	TRIP 8b (Mol + CA1): 1060							
LDR Test: Block 6, Small separation, percent correct to 1st reversal	Supp Fig 1I		Block 6 Small separation % correct to 1st reversal	1-way ANOVA	Treatment*	F (2, 21) = 3.496	**P=0.0489**	SCR vs TRIP8b (Mol): *p=0.0472	0.17	0.00, 0.45
		SCR: 9	SCR: 44.03							
		TRIP8b (Mol): 10	TRIP 8b (Mol): 63.76							
		TRIP8b (Mol + CA1): 5	TRIP 8b (Mol + CA1): 48.46							
LDR Test: Block 6, Small separation, reversal number	Supp Fig 1J		Block 6 Small separation reversal #	1-way ANOVA	Treatment	F (2, 21) = 2.882	P=0.0783	N/A		
		SCR: 9	SCR: 0.3333							
		TRIP8b (Mol): 10	TRIP 8b (Mol): 1.2							
		TRIP8b (Mol + CA1): 5	TRIP 8b (Mol + CA1): 1							

Supplementary Table. Statistical analyses for each figure panel and all results.

Bold text and ***, p<0.05. Italicized text, 0.05<p<0.1. *Magnitudes of partial omega-squared (for RM two-way ANOVA): 0.01 small; 0.06 medium; 0.14 large. N/A not applicable.

**Dependent Measure**	**Figure Panel**	**Group: Subject Number**	**Mean**	**Statistical Test**	**Variables, Main Effect, Interaction** *P<0.5 **P<0.01 ***P<0.001 ****P<0.0001	**F Value**	**P value**	**Post hoc Test** (Bonferroni for 2-way ANOVA, Tukey for 1-way ANOVA)	**Effect size** (when RM two-way ANOVA p<0.05, partial omega-squared is calculated where ≤0.05 small, ≥0.06 medium, ≥0.14 large)	**CI (95%)**
